# Unveiling chlorpyrifos mineralizing and tomato plant-growth activities of *Enterobacter* sp. strain HSTU-ASh6 using biochemical tests, field experiments, genomics, and *in silico* analyses

**DOI:** 10.3389/fmicb.2022.1060554

**Published:** 2022-11-29

**Authors:** Md. Azizul Haque, Md. Shohorab Hossain, Iqrar Ahmad, Md. Ahedul Akbor, Aminur Rahman, Md. Serajum Manir, Harun M. Patel, Kye Man Cho

**Affiliations:** ^1^Department of Biochemistry and Molecular Biology, Hajee Mohammad Danesh Science & Technology University, Dinajpur, Bangladesh; ^2^Division of Computer Aided Drug Design, Department of Pharmaceutical Chemistry, R. C. Patel Institute of Pharmaceutical Education and Research, Shirpur, India; ^3^Institute of National Analytical Research and Services, Bangladesh Council of Scientific and Industrial Research, Dhaka, Bangladesh; ^4^Department of Biomedical Sciences, College of Clinical Pharmacy, King Faisal University, Al Hofuf, Saudi Arabia; ^5^Institute of Radiation and Polymer Technology, Bangladesh Atomic Energy Research Establishment, Dhaka, Bangladesh; ^6^Department of GreenBio Science and Agri-Food Bio Convergence Institute, Gyeongsang National University, Jinju, South Korea

**Keywords:** chlorpyrifos mineralization, *Enterobacter* sp., tomato growth and yield, GC–MS/MS, MD simulation, *in silico* analysis, organophosphate pesticides (OPPs)

## Abstract

The chlorpyrifos-mineralizing rice root endophyte *Enterobacter* sp. HSTU-ASh6 strain was identified, which enormously enhanced the growth of tomato plant under epiphytic conditions. The strain solubilizes phosphate and grew in nitrogen-free Jensen’s medium. It secreted indole acetic acid (IAA; 4.8 mg/mL) and ACC deaminase (0.0076 μg/mL/h) and hydrolyzed chlorpyrifos phosphodiester bonds into 3,5,6-trichloro-2-pyridinol and diethyl methyl-monophosphate, which was confirmed by Gas Chromatography – Tandem Mass Spectrometry (GC–MS/MS) analysis. *In vitro* and *in silico* (ANI, DDH, housekeeping genes and whole genome phylogenetic tree, and genome comparison) analyses confirmed that the strain belonged to a new species of *Enterobacter*. The annotated genome of strain HSTU-ASh6 revealed a sets of nitrogen-fixing, siderophore, *acd*S, and IAA producing, stress tolerance, phosphate metabolizing, and pesticide-degrading genes. The 3D structure of 28 potential model proteins that can degrade pesticides was validated, and virtual screening using 105 different pesticides revealed that the proteins exhibit strong catalytic interaction with organophosphorus pesticides. Selected docked complexes such as α/β hydrolase–crotoxyphos, carboxylesterase–coumaphos, α/β hydrolase–cypermethrin, α/β hydrolase–diazinon, and amidohydrolase–chlorpyrifos meet their catalytic triads in visualization, which showed stability in molecular dynamics simulation up to 100 ns. The foliar application of *Enterobacter* sp. strain HSTU-ASh6 on tomato plants significantly improved their growth and development at vegetative and reproductive stages in fields, resulting in fresh weight and dry weight was 1.8–2.0-fold and 1.3–1.6-fold higher in where urea application was cut by 70%, respectively. Therefore, the newly discovered chlorpyrifos-degrading species *Enterobacter* sp. HSTU-ASh6 could be used as a smart biofertilizer component for sustainable tomato cultivation.

## Introduction

Several strategies have been implemented in the agricultural sector to increase the production of crops/foods, such as the application of biofertilizers, chemical fertilizers, and pesticides, among which synthetic pesticides effectively improve the productivity of food and other agricultural commodities (Popp et al., 2013). Organophosphate (OP) pesticides are highly toxic chemicals that demonstrate extensive activity against pests and comprise approximately 38% of the all pesticides used on crops globally (Zhang et al., 2009). Excessive and inappropriate use of agrochemicals such as pesticides, herbicides, and fertilizers causes severe environmental and health issues (Dar et al., 2019).

To maintain sustainable food production, environmentally protected approaches, biofertilizers are urgently needed to improve crop growth, nitrogen fixation, and decrease yield loss under diverse stress conditions. The utilization of plant growth-promoting (PGP) endophytic bacteria is an efficient approach for stabilizing and improving crop yield. Due to their direct contact to plants, endophytic bacteria may exhibit greater ecological advantages than rhizospheric and epiphytic bacteria (James, 2000). The growth promotion of crop plants is regulated by endophytes by providing plant growth regulators, N fixation, phosphate solubilization, and ACC deaminase activity and improving tolerance against plant biotic and abiotic stresses (Carvalho et al., 2014). The application of PGP rhizobacteria (PPGPR) with plant growth-promoting traits is wanted due to its pragmatic, sustainable, and ecofriendly characteristics (Ali et al., 2022; Moon and Ali, 2022). In fact, a lot of ACC deaminase enzyme secreting PGPR was reported to improve crop plants’ growth and development under various abiotic stress conditions (Moon and Ali, 2022). The PGPR can provide nutrients to non-leguminous plants even though they lack nodules, a process known as “associative nitrogen fixation” (Carvalho et al., 2014).

Furthermore, microorganisms play an important role in the detoxification of synthetic chemicals in soil (Bhatt et al., 2020; Rojas-Sánchez et al., 2022) and in the adaptation and defense of host plants by secreting bioactive compounds (Narayanan and Glick, 2022). They consume almost all-natural and synthetic compounds as their source of carbon and energy. Moreover, a significant number of genes such as *opd* (organophosphate degrading), carboxylesterase, *mpd* (methyl parathion degrading), and amidohydrolase, α/β hydrolase and several enzymes are known to be involved in degrading certain organophosphate pesticides (Yang et al., 2006; Parakhia et al., 2014; Lee et al., 2021; Das et al., 2022).

The endophyte *Enterobacter cloacae* have been reported to promote the growth of crop plants, such as rice, groundnut, black gram, and *Brassica napus* (Panigrahi et al., 2020). It has also been shown to establish nutrient transfer symbiosis with banana plants and protect them against the black sigatoka pathogen (Macedo-Raygoza et al., 2019) and exhibit antagonistic activity against *Pythium* damping-off of cucumber (Kazerooni et al., 2020). However, the effects of *E. cloacae* on tomato plants under epiphytic conditions have not been reported. Tomato plants are susceptible to diseases such as *Fusarium* and *Verticillium* wilt, early and late blight, bacterial speck, and anthracnose. Therefore, several agrochemicals and pesticides are being applied in tropical countries such as Bangladesh to prevent the frequent attacks of pests, insects, and pathogens, which has resulted in the contamination of tomato fruit bodies with excessive pesticide residues that can be ingested through diet and cause health hazards.

Endophytic effects on tomato plant growth and development with *Enterobacter* sp. were not thoroughly investigated. Endophytic effects on tomato plant growth and development with *Enterobacter* sp. were not thoroughly investigated yet. However, several studies have been conducted with other endophytes, such as indole acetic acid (IAA)-secreting *Bacillus subtilis* (Khan et al., 2016), ACC (1-aminocyclopropane-1-carboxylate) deaminase producing *Burkholderia* species (Onofre-Lemus et al., 2009), as well as *Ampelomyces* sp. and *Penicillium* sp. (Morsy et al., 2020), which improved tomato plant growth and promotion. Because tomato plants are non-leguminous, nitrogen fixation is not possible as it is in other leguminous plants. We chose an endophytic bacterium from a non-leguminous rice plant for this purpose. A selective powerful strain namely *Enterobacter* sp. HSTU-ASh6 expressing higher plant growth promoting (PGP) traits such as germination induction, root and shoot development, IAA, ACC-deaminase producing activity, and chlorpyrifos pesticide degrading capability was assessed as biofertilizer components in fields at epiphytic conditions. With the application of this strain in fields, tomato plants demonstrated its resistance against pathogens and pests. Therefore, no additional agrochemicals were used in the fields, resulting in survival of the plants against biotic stress and saving the tomato plant from being impregnated with pesticides. The results of this study will be highly significant for the production of agrochemical-free edible crops with higher yields at a lower cost.

## Materials and methods

### Isolation and biochemical characterization of endophytic strain

The endophytic strain HSTU-ASh6 was isolated from healthy fresh rice plant roots. Two months old rice plants were collected from farmers’ fields near Basherhat (25° 37′ 59.88″ N; 88° 39′ 0.00″ E), Sadar, Dinajpur, Bangladesh. The roots of rice plants were separated from whole plants. To get rid of soil and dust, the root samples were gently washed in distilled water. Following this, twenty root samples were then surface sterilized with 75% ethanol for 3 min and shaken in a 2.5% (w/v) NaOCl solution for 10 min at 120 rpm, which was adopted from Khan et al. (2016) and Das et al. (2022). The root samples underwent additional washings with autoclaved distilled water while they were agitated for 20 min. The roots were rolled on nutrient agar plates, and a 0.1 mL aliquot from the final wash was inoculated into 10 mL of nutrient broth for sterility testing. Samples were discarded if any growth of bacteria was detected in the sterility check (Das et al., 2022). Finally, sterilized rice plant roots were ground using a mortar and pestle and placed in a sterilized test tube (Haque et al., 2015). The squeezed roots were cultured in chlorpyrifos-enriched minimal nutrient media to isolate the chlorpyrifos mineralizing endophytic bacteria, as described (Das et al., 2022). Selected bacteria with prompt growth potentialities were sub-cultured in the same medium to obtain pure colonies, as described previously (Das et al., 2022). The isolate was preserved in 80% (v/v) glycerol at –20°C for further analysis. Biochemical tests, including methyl red, Voges–Proskauer, catalase, KOH string, oxidase, triple sugar iron, citrate utilization, motility indole urease, and urease, and individual sugar fermentation tests such as dextrose, lactose, maltose, and sucrose utilization (Adedayo et al., 2004; Das et al., 2022), were performed to facilitate the identification of the bacterium. In addition, the activities of extracellular cellulase, amylase, protease, and xylanase were determined as described elsewhere (Abdullah-Al-Mamun et al., 2022; Das et al., 2022).

### Plant growth promoting activity tests

#### Indole acetic acid test

The quantification method for IAA of *Enterobacter* sp. HSTU-ASh6 was adopted as previous described (Ullah et al., 2013). Briefly, the bacterial suspension was cultured in 7 mL of modified Luria Bertani broth containing 0.01% tryptophan (L-Trp) separately and combined at 37°C for 7 days in a shaking incubator at 120 rpm. Then, 1 mL of each culture was transferred to 1.5-mL Eppendorf tubes and centrifuged at 10,000 rpm. The supernatant was transferred to glass test tubes to which 2 mL of Salkowski reagent (1 mL of 0.5 mL FeCl_3_ to 50 mL of 35% HClO_4_) was added. Then, the mixture was placed in the dark for 120 min. The resultant reddish-colored spectrum was read using spectrophotometer (UV-VIS spectrophotometer) at 530 nm. Finally, the strain HSTU-ASh6 secreted quantity of IAA was calculated using the standard calibration curve equation of pure IAA (Sigma-Aldrich Ltd, St. Louis, MO, USA) prepared separately.

### 1-Aminocyclopropane-1-carboxylate deaminase activity assay

The ACC deaminase activity of the bacterium was determined according to the modified methods of Shaharoona et al. (2006) and Belimov et al. (2015), which measures the amount of α-ketobutyrate produced upon the hydrolysis of ACC. The endophytic bacterium strain *Enterobacter* sp. strain HSTU-ASh6 was grown in Tryptic Soy Broth medium (TSB) for 18 h at 28°C to determine ACC deaminase activity. The cells were then harvested by centrifugation, washed with 0.1 M Tris–HCl (pH 7.5), and incubated for another 18 h in minimal medium containing 3 mM ACC as the sole source of nitrogen. The bacterial cells were collected by centrifugation and suspended in 5 mL of 0.1 moL L^–1^ of Tris–HCl, pH 7.6, and transferred to microcentrifuge tube. The contents of the tubes were centrifuged at 16,000 rpm for 5 min, and supernatant was removed. The pellets were suspended in 2 mL of 0.1 moL L^–1^ Tris HCl, pH 8.5. Next, 30 μL of toluene was added to the cell suspension and vortexed for 30 s. After 200 μL of the toluenized cells were placed in a fresh microcentrifuge tube, 20 μL of 0.5 moL L^–1^ ACC was added to the suspension, vortexed, and then incubated at 30°C for 15 min. Following the addition of 1 mL of 0.56 moL L^–1^ HCl, the mixture was vortexed and centrifuged for 5 min at 13,000 rpm at room temperature. Two mL of the supernatant was vortexed together with 1 mL of 0.56 moL L^–1^ HCl. Thereupon, 2 mL of the 2, 4-dinitrophenylhydrazine reagent (0.2% 2, 4-dinitrophenylhydrazine in 2 moL L^–1^ HCl) was added to the glass tube, and the contents were vortexed and then incubated at 30°C for 30 min. Following the addition and mixing of 2 mL of 2 moL L^–1^ of NaOH, the absorbance of the mixture was measured by using a spectrophotometer at 540 nm. The cell suspension without ACC was used as a negative control and with (NH_4_)_2_SO_4_ (0.2% w/v) as the positive control. The number of μmol of α-ketobutyrate produced by this reaction was determined by comparing the absorbance at 540 nm of a sample to a standard curve of α-ketobutyrate ranging between 10 and 200 μmoL (Honma and Shimomura, 1978; Shaharoona et al., 2006; Belimov et al., 2015).

### Phosphate solubilization test

The phosphate solubilization ability of the isolate was determined qualitatively by streaking a strain on PVK agar media in a Petri plate. The plate was placed at 30°C for 72 h under aerobic conditions. The colonies that could grow and create a zone on the insoluble phosphate media were selected as the phosphate-solubilizing bacteria (Madhusmita et al., 2017).

### Nitrogen fixation test

The nitrogen fixation capability of the endophytic strain HSTU-ASh6 was determined by streaking the strain on completely nitrogen-free Jensen’s media (Sulistiyani and Meliah, 2017). The strain was grown for 5–7 days at 37°C in an incubator under aerobic conditions.

#### DNA extraction, amplification, sequencing, and analysis

The genomic content of the isolate was extracted using a commercial Quick-DNATM Miniprep Kit specific for bacteria (Zymo Research, Irvine, CA, USA), according to the manufacturer’s specification. The 16S rRNA gene was amplified from the extracted DNA by polymerase chain reaction. The bacterial-specific universal forward primer 27F 5′-AGAGTTTGATCCTGGCTCAG-3′ and reverse primer 1492R 5′-TACGGTTACCTTGTTACGACTT-3′ were used in the PCR mixture. The master mix was prepared by adding Taq buffer, dNTPs, MgCl_2_, and nuclease-free water in a PCR tube. The forward and reverse primers, MgCl_2_, template DNA, and Taq DNA polymerase enzyme were added just before loading the sample in the PCR system (Haque et al., 2020; Das et al., 2022). After 36 cycles of PCR steps, the amplicon was stored at –20°C for further analysis. The amplified PCR products were visualized by agarose gel electrophoresis. DNA purification, cycle sequencing, and DNA sequencing of PCR amplicons were performed using a BDT v3 Cycle sequencing kit in a genetic analyzer 3130 (Haque et al., 2015, 2020). Finally, the 16S rRNA sequence was submitted to GenBank on the NCBI online server.

### Effect of *Enterobacter* sp. strain HSTU-ASh6 on tomato plant growth promotion

The *Enterobacter* sp. HSTU-ASh6 effect on tomato plant growth promotion was assessed in two different ways. Firstly, its effect at seed germination stage was performed in laboratory conditions. Secondly, its effect on tomato plant seedlings development to vegetative and reproductive stages was investigated in fields at epiphytic conditions.

### Effect of strain HSTU-ASh6 on tomato seeds germination

#### Seed sterilization and preparation

The tomato seeds were collected from Bangladesh Agricultural Development Corporation nursery. Firstly, the seeds were allowed to sundry. Then seeds were sterilized with 70% ethanol for 1 min and washed several times with sterilized water.

#### Seed bacterization

The sterilized seeds were placed in two sets of sterilized Eppendorf tubes. The first set of tubes contained seeds in 1 mL of bacterial suspension, and the second set contained seeds in 1 mL of distilled water without the strain (control). All tubes were placed in a dark environment for 8 h. After that the seeds were ready for germination.

#### Seed inoculation onto petri dishes

The seeds were sown for germination on Whatman filter paper in Petri dishes. The petri dishes were arranged in a Completely Randomized Design (CRD) with three replications. After 7 days of seed germination, 0.25 mL of bacterial suspension was applied in each petri dish. After 15 days of seed plantation, the following parameters of tomato plants with and without the strain were recorded.

##### Germination percentage

Germination percentage was calculated using the formula GP = Total germinated seeds/Total number of seeds × 100 (Ashraf and Foolad, 2005).

##### Root length and shoot length

Root length and shoot length were measured using a graduated ruler.

##### Vigor index

The vigor index was calculated using the formula Seedling length × germination percentage.

### Effect of HSTU-ASh6 strain on tomato seedlings in field conditions

Tomato seedlings aged 32 days were collected from a local nursery and planted in the research field using a randomized complete block design method. The experiment was divided into the following five treatments to observe the effect of using different concentrations (30–100%) of urea doses and bacterial strains: T1 (30% urea with bacterium), T2 (70% urea with bacterium), T3 (100% urea with bacterium), Tc (100% urea without bacterium), and T0 (no urea and no bacterium/control). The experimental unit area (plot) was 1.80 m^2^ and consisted of four rows measuring 1.32 m in width and 1.37 m in length, and the seedlings were planted using a hand drill method, maintaining a row-to-row distance of 40 cm and a plant-to-plant distance of 10 cm in a line. The plants were irrigated regularly according to requirement. The treatments were applied to the plants after 10, 20, 30, and 40 days of plantation. The root length, shoot length, leaf size, fresh weight, and dry weight were measured two times after 30 and 45 days of plantation.

### Pesticide degradation analysis using GC–MS/MS

Firstly, the growth of the strain was checked in chlorpyrifos-enriched minimal salt broth media, in which chlorpyrifos (1 g/L) was the sole source of carbon (Lee et al., 2021; Das et al., 2022). Next, 5 μL of the stock bacterium was inoculated into the medium and kept for 14 days, after which 5 mL of chlorpyrifos-containing MSM was transferred into a separating funnel. Secondly, 25 mL of distilled water and 5 mL of *n*-hexane were added to the funnel and shaken vigorously for 5–10 min. The desired solvents in the n-hexane layer appeared on the top layer, whereas the unwanted layer remained on the bottom layer. The bottom layer was separated and kept in a bottle. Finally, to examine the biodegradation of chlorpyrifos, the n-hexane with appropriate solvent-containing layer solution was run on Gas Chromatography – Tandem Mass Spectrometry (GC–MS/MS) (Shimadzu QP2010, Japan). The GC–MS/MS instrumentation was set up according to Al Mansur et al. (2018), and following examination of each compound’s mass spectrum were determined using the NIST11 library (Zaman et al., 2021).

### Genomic DNA extraction, library preparation, whole genome sequencing

The preparation of the genomic library of *Enterobacter* sp. HSTU-ASh6 and the sequencing of the complete genome were performed using the Illumina MiniSeq System (Illumina, Inc., San Diego, CA, USA), as described previously (Abdullah-Al-Mamun et al., 2022; Das et al., 2022). After complete genome sequencing, the quality test was conducted using the fast QC analysis and Illumina Base Space sequence analysis Hub. SPAdes assembler version 3.5 was used to assemble the complete genome of *Enterobacter* sp. HSTU-ASh6, and the resulting assembled genome was annotated and analyzed using NCBI Prokaryotic Genome Annotation Pipeline (PGAP) version 4.5, Prokka. The circular genome map of *Enterobacter* sp. HSTU-ASh6 was prepared using CGView Comparison Tool. Next, different types of genes, e.g., those responsible for plant growth promotion (nitrogen fixation, phosphate solubilization, IAA production, ACC deaminase production, and sulfur assimilation), biofilm formation, root colonization (chemotaxis and mortality), pesticide degradation, and stress tolerance (heat shock and cold shock), were predicted from the PGAP file.

### Strain classification

#### Phylogenetic tree construction

A comparative phylogenetic analysis of housekeeping genes, e.g., 16S rRNA, *gyr*B, *rec*A, *rpo*B, and *ton*B of different closely related *Enterobacter* sp. sequences, was performed using the NCBI database. The phylogenetic tree of HSTU-ASh6 whole genome sequence was constructed along with 29 nearest homologs genebank files accumulated from the NCBI database. The REALPHY 1.12 online server was used to construct the complete genome phylogenetic tree, which was modified using the MEGA-X.0 program. Finally, the phylogenetic trees of the housekeeping genes were constructed by the neighbor-joining method using Molecular Evolutionary Genetics Analysis (MEGA-X).

#### Average nucleotide identity and DNA–DNA hybridization

Average nucleotide identity (ANI) and digital DNA–DNA hybridization (DDH) analyses were performed using the JSpecies WS, Type Strain Genome Server (TYGS) and GGDC web server, respectively (Abdullah-Al-Mamun et al., 2022). A total of 15 types of nearest homolog complete genome sequences were utilized for the ANI analysis. For the DDH analysis, 15 different types of genomes were aligned with the HSTU-ASh6 genome.

### Genome comparison

#### Multiple genome sequence alignment and pangenomic analysis

To determine the genetic diversity and similarities of *Enterobacter* sp. HSTU-ASh6, its genome sequence was compared with the genome sequences of six most closely related species, viz., *E. asburiae* (CP007546), *E. bugandensis* 220 (CP039453), *E. chengduensis* (CP043318), *E. cloacae* A1137 (CP021851), *E. roggenkampii* RHBSTW 00695 (CP056168), and *E. sichuanensis* SGAir0282 (CP027986), by pangenomic analysis using GView Server. The genome of *Enterobacter* sp. HSTU-ASh6 was further compared with four recently published nearest homologs, viz., *E. cloacae* A1137 (NZ_CP021851), *E. sichuanensis* SGAir0282 (NZ_CP027986), *E. roggenkampii* Ecl 20 981 (CP048650), and *E. asburiae* ATCC 35953 (CP011863), by multiple genomic analysis using the progressive Mauve software^1^.

### Insecticide degradation demonstrated by enzyme catalytic reaction

#### Homology modeling, protein preparation, and validation

The homology modeling of the protein was performed using the Iterative Threading ASSEmbly Refinement (I-TASSER) Server. Next, the model was subjected to energy minimization using the steepest descent and conjugate gradient techniques to eliminate lower quality contacts among protein atoms. Computations were performed *in vacuo* with GROMOS 96 43B1 parameters set using the Swiss-PDB Viewer. The 3D structures of the protein were saved in .pdb format. The SAVES v6.0 online server was used to check the validation and evaluate the quality of the minimized protein^2^.

### Small molecule/ligand collection and optimization

A total of 105 various types of organophosphorus pesticides, weed killers, and nerve agent small molecules were collected from the PubChem database^3^. After ordering, the ligands were optimized and minimized using the mmff94 force field and the steepest descent algorithm in the PyRx software environment. The 3D structures of small molecules were stored in a file in .sdf format.

### Virtual screening, molecular docking, and visualization

A total of 105 pesticides (small molecules) with 28 valid protein structures were used individually and screened with 105 ligands for virtual screening. Virtual screening and molecular docking were performed using the PyRx software (Dallakyan and Olson, 2015). Protein–ligand complexes were constructed using the Chimera software, and 2D and 3D structure visualization was performed using Discover Studio 2021 Client. The results of virtual screening were displayed by boxplot analysis performed using the Origin Pro 8.0 software. Furthermore, protein and ligand non-bonded interactions were recognized using Discovery Studio 2021 Client. At the time of virtual screening, the grid box sizes were fixed at 61.6411, 61.6072, and 61.8787, which was the maximum size of the grid box, respectively for X, Y, and Z. However, in the case of molecular docking, the grid box sizes were not fixed, and they were dissimilar as for (i) α/β fold hydrolase (GM298_18675) with crotoxyphos, (ii) carboxylesterase with coumaphos, (iii) α/β fold hydrolase (GM298_08590) with cypermethrin, (iv) α/β fold hydrolase (GM298_00815) protein with diazinon, (v) amidohydrolase family protein (GM298_20245) with chlorpyrifos, and the X, Y, and Z sizes were 61.6411, 61.6072, and 61.8787, respectively. The binding affinity of protein with ligands was calculated in kcal/mole for a negative score.

### Molecular dynamics simulation

To determine the potential pesticide-degrading stability of proteins with pesticides, a molecular dynamics simulation was run using the Desmond program (Schrodinger) that has an explicit solvent MD package linked to the OPLS 2005 force field. The protein preparation wizard was used to create the protein-ligand complexes, and the system builder panel was solvated them using the orthorhombic simple point-charge water model. The counter ions Na+ or Cl^–^ were added as necessary to charge and neutralize the solvated system. In order to relax the system to the lowest local energy, minimization tasks were carried out. Next, this model system was run to MD simulation steps using the OPLS 2005 force field parameter (Lee et al., 2021; Patel et al., 2021). The MD simulation was performed up to 100 ns using the NPT ensemble (isothermal-isobaric ensemble, constant temperature, constant pressure, and constant number of particles) at 300 K temperature and 1.013 bar pressure (Jorgensen et al., 1996; Lee et al., 2021). For controlling temperature and pressure, the Noose-Hover chain thermostat algorithm and the Martyna-Tobias-Klein barostat algorithm were employed, respectively. The particle mesh Ewald method was used to measure long-range electrostatic interactions, while the other parameters were left at their default values (Patel et al., 2021). The behavior and interactions between the ligands and protein were examined using the Simulation Interaction Diagram tool. The output.cms file was imported, and root mean square deviation (RMSD) and root mean square fluctuation (RMSF) was chosen to produce the revealed plots.

## Results

### Isolation and biochemical characterization

The biochemical characterization of HSTU-ASh6 is summarized in Supplementary Figure 1. The strain was gram-negative; KOH dissolves the thin peptidoglycan layer of the cell wall of gram-negative bacteria, and they showed viscously and stuck in the loop within 30 s after the addition of KOH to the bacteria cultured for 24 h. Conversely, the isolate demonstrated positive results in the Voges–Proskauer, catalase, triple sugar iron, citrate utilization, motility indole urease, urease, dextrose, lactose, maltose, and sucrose utilization tests. However, the strain showed negative results in the indole, MR, and oxidase tests. It also exhibited mild activities of cellulase, amylase, and xylanase but a vigorous protease activity in the plate assay.

### P solubilization, N fixation, indole acetic acid production, 1-aminocyclopropane-1-carboxylate deaminase activity

The results of phosphate solubilization, nitrogen fixation, and IAA production are summarized in Supplementary Figure 2. The phosphate solubilization test was positive because growth was detected and a hollow zone appeared in the PVK plate. The nitrogen fixation test was also positive based on the detection of the growth of HSTU-ASh6 in Jensen’s medium. Moreover, the strain was capable of producing IAA as demonstrated by the reddish pink color, which amounted to 4.8 mg/mL compared to that in the control. In addition, the ACC deaminase activity of the strain was 0.0076 μg/mL/h.

### Growth promotion

#### Seed germination effect on tomato seeds

The HSTU-ASh6 suspension was applied three times to the tomato seeds sown on a filter paper in a Petri dish. After 9 days, the germination percentage, root length, shoot length, seedling length, and vigor index were recorded as 92%, 6.09, 5.39, 12.02 cm, and 1109.57 in the treated plants, respectively. In the control (seeds not treated with the bacterial strain), the respective values were 69%, 3.50, 5.34, 8.84 cm, and 611.99 (Table 1).

**TABLE 1:** Germination percentage, root length, shoot length, seedling length, and vigor index of tomato plant with the strain at 9 days of seed plantation.

Name of isolate	Germination percentage	Root length (cm)	Shoot length (cm)	Seedling length (cm)	Vigor index
Control	69.23 ± 0.0^b^	3.50 ± 0.58^b^	5.34 ± 0.11^ab^	8.84 ± 0.53^b^	611.99 ± 36.71^b^
HSTU-ASh6 (P7)	92.31 ± 0.0^a^	6.09 ± 0.26^a^	5.39 ± 0.13^a^	12.02 ± 0.28^a^	1109.57 ± 25.58^a^

Different lowercase letters indicate significant difference at the 5% level.

#### Tomato plant growth at vegetative and reproductive stages

The root length, shoot length, leaf length, fresh weight, and dry weight of tomato plants measured after 30 and 45 days of plantation are shown in Table 2. The highest root length, shoot length, leaf length, fresh weight, and dry weight were observed in T3 treatment. In particular, the fresh and dry weights of tomato plants were significantly higher in T3 treatment than in T2 and other treatments. The fresh and dry weights were 1.33–2.93-fold and 1.8–2.43-fold higher in T3 treatment, 1.95–2.3-fold and 1.41–2.17-fold higher in T2 treatment, 1.8–2.0-fold and 1.3–1.6-fold higher in T1 treatment, respectively, than in Tc treatment after 30–45 days of plantation. These results suggest that the inoculation of *Enterobacter* sp. strain HSTU-ASh6 significantly improved tomato plants’ growth and development in fields at epiphytic conditions where the amount of urea fertilizer application was cut by 30–70% (Table 2 and Supplementary Figure 3).

**TABLE 2:** Effect of bacterium and fertilizer in different time period among different treatment group.

Data collection time	Treatments	Root length (cm)	Shoot length (cm)	Leaf length (cm)	Fresh weight (gm)	Dry weight (gm)
After 30 days of plantation	T1	22.33 ± 1.86^ab^	32.67 ± 2.84^a^	20.33 ± 0.88^ab^	150 ± 1.16^c^	78 ± 1.16^c^
	T2	24.33 ± 0.33^a^	34 ± 0.58^a^	21 ± 0.58^a^	162.67 ± 1.45^b^	85.33 ± 0.88^b^
	T3	25.33 ± 0.67^a^	35 ± 0.58^a^	21.33 ± 0.67^a^	180 ± 1.15^a^	90.33 ± 1.20^a^
	Tc	15.33 ± 0.88^c^	27 ± 0.58^b^	18 ± 0.58^c^	83.33 ± 2.01^d^	60.33 ± 1.45^d^
	To	10.67 ± 0.67^d^	22.33 ± 0.88^c^	16 ± 0.58^d^	69.33 ± 1.20^e^	41 ± 1.53^e^
After 45 days of plantation	T1	26.33 ± 1.45^a^	36.67 ± 2.88^a^	22 ± 0.58^ab^	190 ± 1.73^c^	124.67 ± 2.60^c^
	T2	28 ± 1^a^	38 ± 1.53^a^	23.33 ± 0.88^a^	221 ± 2.08^b^	168 ± 1.53^b^
	T3	29 ± 3.06^a^	40.33 ± 0.88^a^	23.67 ± 0.33^a^	278.33 ± 2.12^a^	187.33 ± 1.45^a^
	Tc	17.67 ± 0.67^b^	30.67 ± 2.12^b^	20.67 ± 1.45^c^	95 ± 1.16^d^	77.33 ± 1.45^d^
	To	12 ± 0.58^c^	25 ± 1.15^c^	17.33 ± 0.33^d^	83.67 ± 0.88^e^	56.67 ± 1.20^e^

T1, 30% urea treatment + strain HSTU-ASh6 treatment; T2, 70% urea treatment + HSTU-ASh6 treatment; T3, 100% urea treatment + HSTU-ASh6 treatment; T0, 0% urea treatment; Tc, 100% urea treatment. Different lowercase letters indicate significant difference at the 5% level.

### Chlorpyrifos biodegradation analysis using GC–MS

**FIGURE 1:**
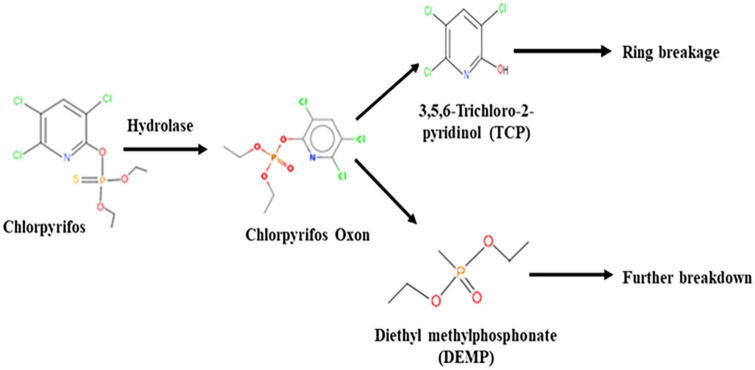
A proposed pathway of chlorpyrifos metabolism by the strain *Enterobacter* sp. HSTU-ASh6.

The GC–MS/MS analysis spectra revealed the presence of phorate sulfoxide, phorate sulfone, chlorpyrifos methyl, carbophenothion sulfoxide, oxydisulfoton, carbonochloridic acid, 2,4,5-trichlorophenyl ester, thionodemeton sulfone, 3-(2-thienyl)-DL-alanine, chlorpyrifos oxon, diethyl methanephosphonate (DEMP), and 3,5,6-trichloro-2-pyridinol (TCP), etc. (Table 3). On the basis of GC–MS/MS results, a pathway of chlorpyrifos biodegradation by HSTU-ASh6 was proposed as illustrated in Figure 1. Chlorpyrifos was initially degraded by hydrolysis to generate chlorpyrifos oxon that was further broken down to generate TCP and DEMP (spectrum = 8). Subsequently, TCP was again degraded by ring breakage, resulting in its complete detoxification.

**TABLE 3:** Spectrum, molecular form and chemical structure of chlorpyrifos (1 gm/100 mL) biodegradation observed using GC–MS/MS analysis.

Similarity of hit	Search Spectrum	Soft ionization (SI)	Spectrum	Molecular weight (Da)	Molecular form	Chemical structure
						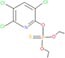
1	77	2921	88	2	Chlorpyrifos	
						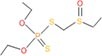
7	60	2588	3	6	Phorate sulfoxide	
						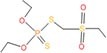
8	59	2588	4	7	Phorate sulfone	
						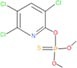
12	56	2598	13	0	Chlorpyrifos-methyl	
						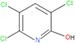
14	55	6515	38	4	2-Hydroxy-3,5,6-trichloropyridine/**3,5,6-Trichloro-2-pyridinol (TCP)**	
						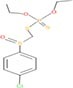
17	54	17297	40	4	Carbofenothion sulfoxide	
						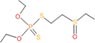
19	53	2497	7	6	Oxydisulfoton	
						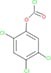
20	53	16947	69	6	Carbonochloridic acid, 2,4,5-trichlorophenyl ester	
						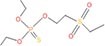
21	52	4891	54	7	Thionodemeton sulfone	
						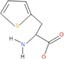
22	52	2021	58	1	3-(2-Thienyl)-dl-alanine	
						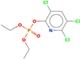
23	51	5598	15	2	Phosphoric acid, diethyl 3,5,6-trichloro-2-pyridyl ester/**Chlorpyrifos oxon**	
						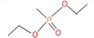
24	51	683	8	9	Diethyl methanephosphonate	

### Genomic features of *Enterobacter* sp. HSTU-ASh6

**FIGURE 2:**
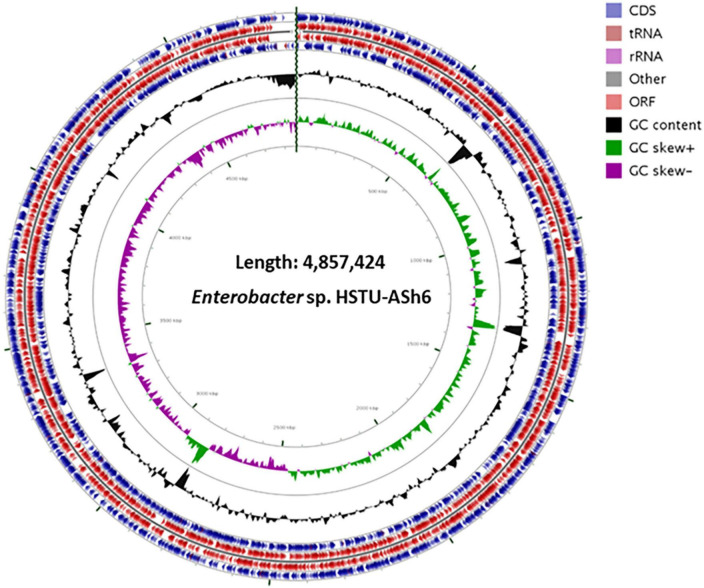
CDS map of the annotated genome of *Enterobacter* sp. HSTU-ASh6.

The genome size of *Enterobacter* sp. HSTU-ASh6 was 4,857,424 bp with 55.1% GC, 4478 protein-coding sequences (CDS), and 72 tRNAs. The subsystem of the RAST annotation is shown in Supplementary Figure 4. All proteins of *Enterobacter* sp. HSTU-ASh6 were revealed by CGview analysis in a proper genomic map (Figure 2). The genomic map of proteins from *Enterobacter* sp. HSTU-ASh6 was created successfully, where GC-skew positivity indicates the presence of CDS downstream and GC-skew negativity shows the presence of CDS upstream. Although GC-skew is predominantly introduced by RNA synthesis in local genomic areas, it was first used to estimate the *ori* and *ter* positions computationally by looking at known genome sequences.

### Taxonomic classification

#### Phylogenetic tree

##### Phylogenetic tree of housekeeping genes

**FIGURE 3:**
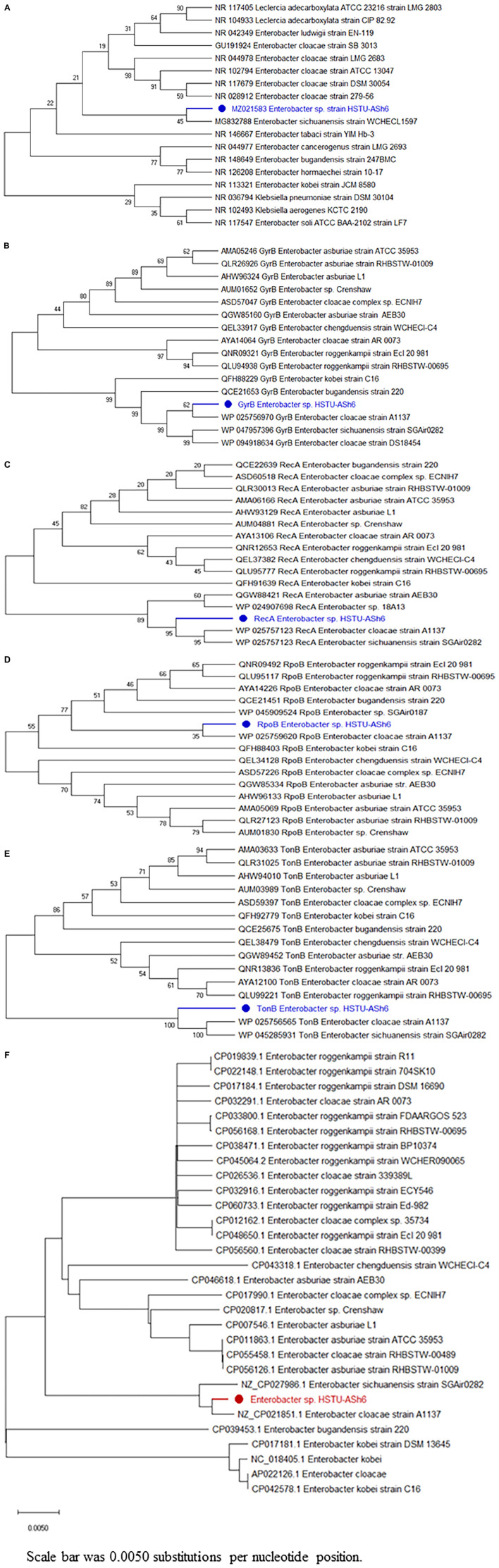
Phylogenetic tree constructed using the housekeeping genes of HSTU-ASh6. **(A)** 16S rRNA, **(B)** gyrase B, **(C)** RecA, **(D)** rpoB, **(E)** tonB, and **(F)** whole genome phylogenetic tree of *Enterobacter* sp. HSTU-ASh6.

The 16S rRNA phylogenetic tree demonstrated that HSTU-ASh6 positioned in the same sister taxa (with 45% similarity) with *E. sichuanensis* WCHECL1597 (Figure 3A). According to *gyr*B (Figure 3B) and *rpo*B (Figure 3C) alignment tree, HSTU-ASh6 was located in the same sister taxa with *E. cloacae* A1137 with 62% and 35% similarities, respectively. Moreover, *rec*A (Figure 3D) tree showed that HSTU-ASh6 was located in a different clade with *E. cloacae* A1137 and *E. sichuanensis* WCHECL1597. Moreover, *tonB* (Figure 3E) phylogenetic tree showed that HSTU-ASh6 was placed with *E. cloacae* A1137 and *E. sichuanensis* WCHECL1597 in the different taxa of the same node. In particular, HSTU-ASh6 was placed in the same node and was more close to *E. cloacae* A1137 in the whole genome sequence phylogenetic tree (Figure 3F).

#### Genome comparison

The pairwise ANI blast (ANIb) value observed between HSTU-ASh6 and *E. cloacae* A1137 was 98.89%. In fact, *E. sichuanensis* SGAir0282, *E. roggenkampii* RHBSTW, *E. asburiae* RHBSTW-01009, and *E. roggenkampii* Ecl 20 981 showed 98.12, 91.12, 91.02, and 91.06% ANIb values, respectively, where other *Enterobacter* species showed ANIb values < 91% (Supplementary Table 1). The DNA–DNA hybridization analysis of HSTU-ASh6 with its nearest homolog *E. cloacae* A1137 showed 92.6% DDH, whereas all other *Enterobacter* strains showed 38.9–45.6% DDH, except *E. sichuanensis* SGAir0282 that showed 85.6% DDH (Table 4).

**TABLE 4:** Digital DNA–DNA hybridization (dDDH) for species determination of *Enterobacter* sp. HSTU-ASh6 depends on whole genome sequences.

Subject strain	dDDH (d0, in %)	C.I. (d0, in%	DDH (d4, in%)	C.I. (d4, in%)	dDDH (d6, in %)	C.I. (d6, in%)	G + C content difference (in %)
*Enterobacter sichuanensis* WCHECL1597	85.8	[82.1– 88.8]	85.8	[83.2– 88.1]	88.7	[85.8– 91.0]	0.12
*Enterobacter roggenkampii* DSM16690	74.1	[70.1– 77.7]	45.4	[42.9– 48.0]	69.1	[65.7– 72.3]	0.92
*Enterobacter asburiae* ATCC 35953	67.8	[63.9– 71.5]	45.1	[42.5– 47.7]	63.9	[60.6– 67.1	0.34
*Enterobacter quasiroggenkampii* WCHECL1060 T	75.5	[71.5– 79.1]	44.6	[42.1– 47.2]	69.9	[66.4– 73.1	0.56
*Enterobacter vonholyi* E13T	76.3	[72.3– 79.9]	44.1	[41.6– 46.7]	70.3	[66.9– 73.6]	0.45
*Enterobacter dykesii* E1T	75.5	[71.5– 79.0]	44.0	[41.5– 46.6]	69.6	[66.2– 72.9]	0.72
*Enterobacter chengduensis* WCHECl-C4	67.2	[63.3– 70.8]	42.5	[40.0– 45.1]	62.4	[59.1– 65.6]	0.61
*Enterobacter bugandensis* EB-247	75.7	[71.7– 79.3]	39.8	[37.4– 42.2]	68.0	[64.5– 71.2]	0.87
*Enterobacter chuandaensis* 090028T	73.6	[69.6– 77.2]	39.7	[37.2– 42.2]	66.2	[62.8– 69.5]	0.56
*Enterobacter kobei* DSM 13645	65.5	[61.7– 69.2]	39.2	[36.7– 41.7]	59.8	[56.5– 62.9	0.22
*Enterobacter quasimori* 090044	71.0	[67.1– 74.7]	36.4	[34.0– 38.9]	62.6	[59.3– 65.8]	0.64
*Enterobacter ludwigii* DSM 16688	70.1	[66.2– 73.8]	36.6	[32.2– 37.2]	61.0	[57.8– 64.2]	0.53
*Enterobacter cancerogenus* ATCC33241	56.6	[53.0– 60.1]	31.2	[28.8– 33.7]	49.5	[46.4– 52.5	0.56
*Leclercia adecarboxylata* NBRC102595	44.0	[40.7– 47.5	25.8	[23.5– 28.3]	38.2	[35.2– 41.2	0.44
*Enterobacter kobei* ATCC BAA-260	33.2	[29.8– 36.7]	22.5	[20.2– 25.0]	29.4	[26.5– 32.5]	0.33

The strain comparison was done in type strain genome server (TYGS).

**FIGURE 4:**
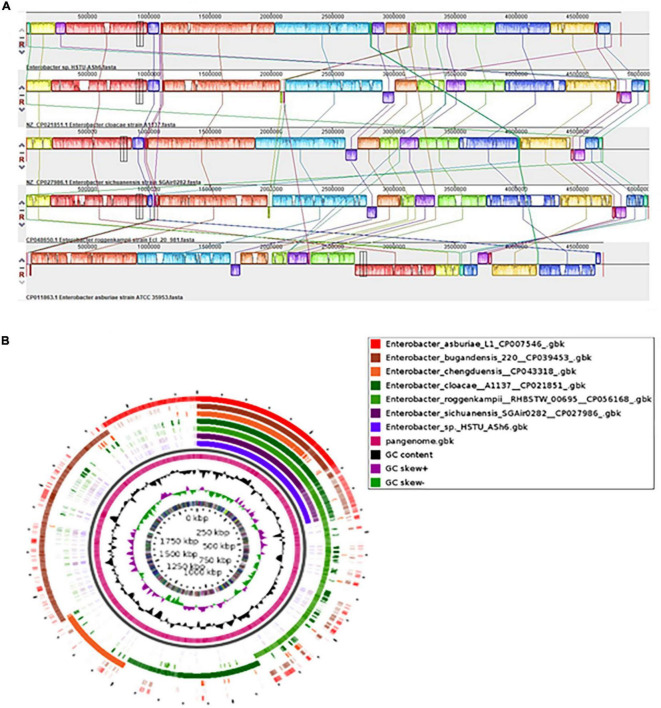
**(A)** Synteny analysis of *Enterobacter* sp. HSTU-ASh6 strain with other close related strains. **(B)** Pangenomic comparison map of *Enterobacter* sp. HSTU-ASh6 with nearest homologs. Multiple genome sequence analysis of *Enterobacter* sp. HSTU-ASh6 with other closely related strains. The same color module represents the homologs region. Good homology of *Enterobacter* sp. HSTU-ASh6 and other *E. cloacae* strains is shown.

The locally collinear block (LCB) of HSTU-ASh6 genome did not completely match with the LCBs of genomes included for genomic comparison. Therefore, there was a rearrangement of genomic regions between the two genomes in terms of collinearity. As shown in Figure 4A, HSTU-ASh6 shared the highest homologous region with A1137. It also shared almost similar LCBs as those of A1137 strain with SGAir0282. We performed a pangenomic analysis of HSTU-ASh6 with six nearest homologs to further investigate the genomic variance. In the circular plot, the pink color slot is identified as the pangenome, and the white space indicates a region missing in the specified genome (Figure 4B). The circular plot clearly indicates that several portions of the genome sequence of HSTU-ASh6 were not similar compared with the other six closest strains.

### Plant growth-promoting and stress-tolerating genes

The genome of *Enterobacter* sp. HSTU-ASh6 harbors various types of PGP genes, namely those encoding nitrogen fixation (*isc*U, *nif*J, *isc*A, *isc*R, *isc*S, *isc*X, *suf*ABCDES, and *fdx*), nitrosative stress (*ntr*B, *nor*R, *nor*V, *nsr*R, and *gln*K), nitrogen metabolism regulation (*gln*D, *gln*B, and *pts*N), ammonia assimilation (*glt*B), ACC deaminase (*dcy*D and *rim*M), siderophore enterobactin (*fes*, *ent*FSD, *fhu*ABCD, and *tonB*), IAA production (*trp*CFBDS and *ipd*C), phosphate metabolism (*pit*A, *pst*ABCS, *phoU*, *ugpABE*, *pho*ABERHQ, and *pnt*AB), biofilm formation (*tom*B, *luxS*, *efp*, *flg*ABCDGHIJKLMN, *mot*AB, and *hfq*), sulfur assimilation (*cys*ACHIJKMNTWZ), sulfur metabolism (*fdx*H and *cys*ACDEHIJKMNQSWZ), root colonization (*che*ZYBRWA, *malE*, *rbsB*, *fli*ZDSTFZGHIJKMPQR, *hof*C, *pga*ABCD, *mot*AB, and *mur*J), superoxide dismutase (*sod*ABC), and trehalose metabolism (*treBCR*, *ots*AB, and *lamb*) (Table 5). Furthermore, numerous types of genes associated with stress tolerance e.g., heat shock (*smp*B, *ibp*AB, *hsp*Q, *dna*JK, *rpo*H, *lep*A, and *grp*E), cold shock (*csp*ADE), and drought resistance (*nha*A, *cha*AB, *pro*ABQVWXPS, *bet*ABT, *trk*AH, and *kdp*ABCF) were detected in the HSTU-ASh6 genome (Table 5). These genes are essential for the plant to survive and grow in a harsh environment.

**TABLE 5:** Genes involved in plant growth promoting, stress tolerating, and pesticide degrading activity.

PGP activities description	Gene name	Locus tag	CDS	Product	E.C. number
Nitrogen fixation	*isc*U	GM298_14895	87842.88228	Fe–S cluster assembly scaffold *IscU*	–
	*nif*J	GM298_00740	154364.157888	Pyruvate: ferredoxin (flavodoxin) oxidoreductase	–
	*isc*A	GM298_14890	87505.87828	Iron–sulfur cluster assembly protein IscA	–
	*isc*R	GM298_14905	89587.90078	Fe–S cluster assembly transcriptional regulator IscR	–
	*isc*S	GM298_14900	88253.89467	IscS subfamily cysteine desulfurase	2.8.1.7
	*suf*A	GM298_13615	3786.4154	Fe–S cluster assembly scaffold SufA	–
	*suf*B	GM298_13620	4163.5653	Fe–S cluster assembly protein SufB	–
	*suf*C	GM298_13625	5663.6409	Fe–S cluster assembly ATPase SufC	–
	*suf*D	GM298_13630	6384.7655	Fe–S cluster assembly protein SufD	–
	*suf*S	GM298_13635	7652.8872	Cysteine desulfurase SufS	2.8.1.7
	*suf*E	GM298_13640	8887.9303	Cysteine desulfuration protein SufE	–
	*fdx*	GM298_14875	84707.85042	ISC system 2Fe–2S type ferredoxin	–
	*isc*X	GM298_14870	84505.84705	Fe–S cluster assembly protein IscX	–
	*hsc*A	GM298_14880	85044.86894	Fe–S protein assembly chaperone HscA	–
	*hsc*B	GM298_14885	GM298_14885	Co-chaperone HscB	–
Nitrosative stress	*ntr*B	GM298_17410	55851.56729	Nitrate ABC transporter permease	–
	*nor*R	GM298_06640	225394.226908	Nitric oxide reductase transcriptional regulator *NorR*	–
	*nor*V	GM298_06635	223761.225206	Anaerobic nitric oxide reductase flavorubredoxin	–
	*nsr*R	GM298_16725	41824.42249	Nitric oxide-sensing transcriptional repressor NsrR	–
	*gln*K	GM298_11020	192488.192826	P-II family nitrogen regulator	–
Nitrogen metabolism regulatory protein	*gln*D	GM298_21660	33792.36467	Bifunctional uridylyltransferase GlnD	2.7.7.59
	*gln*B	GM298_14955	99618.99956	Nitrogen regulatory protein P-II	–
	*pts*N	GM298_07545	78409.78900	PTS IIA-like nitrogen regulatory protein PtsN	–
Ammonia assimilation	*glt*B	GM298_07465	54616.59076	Glutamate synthase large subunit	1.4.1.13
ACC deaminase	*dcy*D	GM298_02800	122850.123836	D-cysteine desulfhydrase	4.4.1.15
	*rim*M	GM298_07110	315522.316061	Ribosome maturation factor *RimM*	–
**Siderophore**					
Siderophore enterobactin	*fes*	GM298_16460	106085.107284	Enterochelin esterase	3.1.1.-
	*ent*F	GM298_16470	107504.111361	Enterobactin non-ribosomal peptide synthetase *EntF*	6.3.2.14
	*ent*S	GM298_16490	114337.115584	Enterobactin transporter EntS	–
	*ent*D	GM298_16450	102997.103638	Enterobactin synthase subunit *EntD*	6.3.2.14
	*fhu*A	GM298_08850	13884.16133	FerrichromeporinFhuA	–
	*fhu*B	GM298_08835	10167.12149	Fe (3+)-hydroxamate ABC transporter permease *FhuB*	–
	*fhu*C	GM298_08845	13036.13833	Fe3+ -hydroxamate ABC transporter ATP-binding protein *FhuC*	–
	*fhu*D	GM298_08840	12146.13036	Fe(3 +)-hydroxamate ABC transporter substrate-binding protein *FhuD*	-
	*ton*B	GM298_00165	26191.26913	TonB system transport protein *TonB*	–
	*fep*B	GM298_20350	112.1071	Fe2 + -enterobactin ABC transporter substrate-binding protein	–
	*fep*G	GM298_16480	112235.113224	Iron-enterobactin ABC transporter permease	–
	*exb*B	GM298_08520	272764.273495	Tol-pal system-associated acyl-CoA thioesterase	–
**Plant hormones**					
IAA production	*trp*CF	GM298_00215	33816.35174	Bifunctional indole-3-glycerol-phosphate synthase TrpC/phosphoribosylanthranilate isomerase TrpF	4.1.1.48/5.3.1.24
	*trp*S	GM298_19565	34038.35042	Tryptophan–tRNA ligase	6.1.1.2
	*trp*B	GM298_00210	32612.33805	Tryptophan synthase subunit beta	4.2.1.20
	*trp*D	GM298_00220	35178.36773	Bifunctional anthranilate synthase glutamate amidotransferase component TrpG/anthranilate phosphoribosyltransferaseTrpD	2.4.2.18/4.1.3.27
	*ipd*C	GM298_13340	183015.184673	Indolepyruvate decarboxylase	4.1.1.74
Phosphate metabolism	*pit*A	GM298_20710	16363.17862	Inorganic phosphate transporter PitA	–
	*pst*S	GM298_11455	21647.22687	Phosphate ABC transporter substrate-binding protein PstS	–
	*pst*C	GM298_11460	22816.23775	Phosphate ABC transporter permease PstC	–
	*pst*A	GM298_11465	23775.24665	Phosphate ABC transporter permease PstA	–
	*pst*B	GM298_11470	24713.25486	Phosphate ABC transporter ATP-binding protein PstB	–
	*pho*U	GM298_11475	25513.26238	Phosphate signaling complex protein PhoU	3.5.2.6
	*ugp*A	GM298_18020	82211.83098	sn-glycerol-3-phosphate ABC transporter permease UgpA	–
	*ugp*B	GM298_18025	83268.84584	sn-glycerol-3-phosphate ABC transporter substrate-binding protein UgpB	–
	*ugp*E	GM298_18015	81369.82214	sn-glycerol-3-phosphate ABC transporter permeaseugpE	–
	*pho*A	GM298_10665	117286.118701	Alkaline phosphatase	–
	*pho*E	GM298_10440	67233.68285	PhosphoporinPhoE	–
	*pho*B	GM298_10730	130482.131171	Phosphate response regulator transcription factor PhoB	–
	*pho*R	GM298_10735	131193.132488	Phosphate regulon sensor histidine kinase PhoR	2.7.13.3
	*pho*H	GM298_15155	2924.3988	Phosphate starvation-inducible protein PhoH	–
	*pnt*A	GM298_14045	87292.88821	Re/Si-specific NAD(P)(+) transhydrogenase subunit alpha	1.6.1.2
	*pnt*B	GM298_14050	88832.90220	Re/Si-specific NAD(P)(+) transhydrogenase subunit beta	–
	*pho*Q	GM298_15690	106230.107693	Two-component system sensor histidine kina se PhoQ	2.7.13.3
Biofilm formation	*tom*B	GM298_11140	213029.213403	Hha toxicity modulator TomB	–
	*lux*S	GM298_06765	245170.245685	*S*-ribosylhomocysteinelyase	4.4.1.21
	*efp*	GM298_16560	11964.12530	Elongation factor P	–
	*flg*A	GM298_02415	43673.44368	Flagellar basal body P-ring formation protein FlgA	–
	*flg*B	GM298_02420	44617.45027	Flagellar basal body rod protein FlgB	–
	*flgC*	GM298_02425	45034.45438	Flagellar basal body rod protein FlgC	–
	*flg*D	GM298_15380	39257.39967	Flagellar hook assembly protein FlgD	–
	*flg*G	GM298_15395	42032.42814	Flagellar basal-body rod protein FlgG	–
	*flg*H	GM298_15400	42872.43570	Flagellar basal body L-ring protein FlgH	–
	*flg*I	GM298_15405	43583.44680	Flagellar basal body P-ring protein FlgI	–
	*flg*J	GM298_15410	44680.45633	Flagellar assembly peptidoglycan hydrolase FlgJ	3.2.1.-
	*flg*K	GM298_15415	45709.47349	Flagellar hook-associated protein FlgK	–
	*flg*L	GM298_15420	47364.48317	Flagellar hook-filament junction protein FlgL	–
	*flg*N	GM298_15355	36787.37212	Flagella biosynthesis chaperone FlgN	–
	*flg*M	GM298_15360	37217.37510	Anti-sigma-28 factor FlgM	–
	*mot*A	GM298_02495	58314.59210	Flagellar motor stator protein MotA	–
	*mot*B	GM298_02500	59207.60112	Flagellar motor protein MotB	–
	*hfq*	GM298_16695	35967.36278	RNA chaperone Hfq	–
Sulfur assimilation	*cys*Z	GM298_13450	201004.201765	Sulfate transporter CysZ	–
	*cys*K	GM298_13455	201929.202900	Cysteine synthase A	2.5.1.47
	*cys*M	GM298_13480	206684.207595	Cysteine synthase CysM	2.5.1.47
	*cys*A	GM298_13485	207714.208808	Sulfate/thiosulfate ABC transporter ATP-binding protein CysA	–
	*cys*W	GM298_13490	208798.209673	Sulfate/thiosulfate ABC transporter permease CysW	–
	*cys*C	GM298_06350	167077.167682	Adenylyl-sulfate kinase	2.7.1.25
	*cys*N	GM298_06345	165653.167077	Sulfate adenylyltransferase subunit CysN	2.7.7.4
	*cys*D	GM298_06340	164735.165643	Sulfate adenylyltransferase subunit CysD	2.7.7.4
	*cys*H	GM298_06325	161245.161979	Phosphoadenosinephosphosulfate reductase	1.8.4.8
	*cys*I	GM298_06320	159433.161145	Assimilatory sulfite reductase (NADP) hemoprotein subunit	1.8.1.2
	*cys*J	GM298_06315	157628.159433	NADPH-dependent assimilatory sulfite reductase flavoprotein subunit	1.8.1.2
	*cys*T	GM298_13495	209673.210506	Sulfate/thiosulfate ABC transporter permease CysT	–
Sulfur metabolism	*cys*C	GM298_06350	167077.167682	Adenylyl-sulfate kinase	2.7.1.25
	*cys*N	GM298_06345	165653.167077	Sulfate adenylyltransferase subunit CysN	2.7.7.4
	*cys*D	GM298_06340	164735.165643	Sulfate adenylyltransferase subunit CysD	2.7.7.4
	*cys*H	GM298_06325	161245.161979	Phosphoadenosinephosphosulfate reductase	1.8.4.8
	*cys*I	GM298_06320	159433.161145	Assimilatory sulfite reductase (NADPH) hemoprotein subunit	1.8.1.2
	*cys*J	GM298_06315	157628.159433	NADPH-dependent assimilatory sulfite reductase flavoprotein subunit	1.8.1.2
	*cys*E	GM298_12115	152433.153254	Serine *O*-acetyltransferase	2.3.1.30
	*cys*Q	GM298_16885	72750.73490	3′(2′),5′’-bisphosphate nucleotidase CysQ	3.1.3.7
	*cys*K	GM298_13455	201929.202900	Cysteine synthase A	2.5.1.47
	*cys*S	GM298_11355	260870.262255	Cysteine–tRNA ligase	6.1.1.16
	*cys*Z	GM298_13450	201004.201765	Sulfate transporter CysZ	–
	*cys*M	GM298_13480	206684.207595	Cysteine synthase CysM	2.5.1.47
	*cys*A	GM298_13485	207714.208808	Sulfate/thiosulfate ABC transporter ATP-binding protein CysA	–
	*cys*W	GM298_13490	208798.209673	Sulfate/thiosulfate ABC transporter permease *CysW*	–
	*fdx*H	GM298_01155	243317.244198	Formate dehydrogenase subunit beta	–
Antimicrobial peptide	*pag*P	GM298_20535	36991.37608	Lipid IV(A) palmitoyltransferase PagP	2.3.1.251
	*sap*B	GM298_00400	75872.76837	Peptide ABC transporter permease *SapB*	–
**Root colonization**					
Chemotaxis	*che*Z	GM298_02265	17971.18609	Protein phosphatase CheZ	3.6.1.-
	*che*Y	GM298_02270	18615.19001	Chemotaxis protein CheY	-
	*che*B	GM298_02275	18991.20070	Chemotaxis-specific protein-glutamate methyltransferase *CheB*	3.1.1.61
	*che*R	GM298_03355	215588.216454	Protein-glutamate *O*-methyltransferase CheR	2.1.1.80
	*che*W	GM298_03310	204215.204718	Chemotaxis protein CheW	–
	*che*A	GM298_02505	60099.62018	Chemotaxis protein CheA	–
	*mal*E	GM298_21870	9111.10301	Maltose/maltodextrin ABC transporter substrate-binding protein MalE	–
	*rbs*B	GM298_22990	3204.4094	Ribose ABC transporter substrate-binding protein RbsB	–
Motility	*fli*Z	GM298_02790	121304.121855	Flagella biosynthesis regulatory protein FliZ	–
	*Fli*D	GM298_02755	111757.113181	PRJNA591446:GM298_02755	–
	*fli*S	GM298_02750	111331.111735	Flagellar export chaperone FliS	–
	*fli*T	GM298_02745	110951.111325	Flagella biosynthesis regulatory protein FliT	–
	*fli*F	GM298_02290	21526.23208	Flagellar basal body M-ring protein FliF	–
	*fli*E	GM298_02730	108072.108386	Flagellar hook-basal body complex protein FliE	–
	*fli*G	GM298_02720	105176.106174	Flagellar motor switch protein FliG	–
	*fli*H	GM298_02715	104476.105183	Flagellar assembly protein FliH	–
	*fli*I	GM298_02710	103106.104476	Flagellum-specific ATP synthase FliI	–
	*Fli*J	GM298_02705	102641.103084	Flagella biosynthesis chaperone FliJ	–
	*fli*K	GM298_02700	101409.102644	Flagellar hook length control protein FliK	–
	*fli*M	GM298_02325	28414.29406	Flagellar motor switch protein FliM	–
	*fli*P	GM298_02675	98299.99036	Flagellar type III secretion system pore protein FliP	–
	*fli*Q	GM298_02670	98020.98289	Flagellar biosynthesis protein FliQ”	–
	*fli*R	GM298_02665	97227.98012	Flagellar type III secretion system protein FliR	–
**Adhesive structure**	*hof*C	GM298_09075	69182.70366	Protein transport protein HofC	–
**Adhesin production**	*pga*A	GM298_08655	298669.301107	Poly-beta-1,6-*N*-acetyl-D-glucosamine export porin PgaA	–
	*pga*B	GM298_08650	296723.298660	Poly-beta-1,6-*N*-acetyl-D-glucosamine *N*-deacetylase PgaB	–
	*pga*C	GM298_08645	295399.296730	Poly-beta-1,6-*N*-acetyl-D-glucosamine synthase	–
	*pga*D	GM298_08640	294968.295402	Poly-beta-1,6-*N*-acetyl-D-glucosamine biosynthesis protein PgaD	–
**Flageller protein**	*fli*P	GM298_02675	98299.99036	Flagellar type III secretion system pore protein FliP	–
	*mot*A	GM298_02495	58314.59210	Flagellar motor stator protein MotA	–
	*mot*B	GM298_03300	201225.202154	Flagellar motor stator protein MotB	–
	*mur*J	GM298_15350	35192.36727	Murein biosynthesis integral membrane protein murJ	–
**Superoxide dismutase**	*sod*A	GM298_18770	40834.41454	Superoxide dismutase [Mn]	1.15.1.1
	*sod*B	GM298_13795	37254.37835	Superoxide dismutase [Fe]	1.15.1.1
	*sod*C	GM298_13845	45550.46068	Superoxide dismutase [Cu–Zn] SodC2	1.15.1.1
**Trehalose metabolism**	*tre*B	GM298_21730	11825.13243	PTS trehalose transporter subunit IIBC	2.7.1.201
	*tre*C	GM298_21725	10130.11773	Alpha, alpha-phosphotrehalase	3.2.1.93
	*tre*R	GM298_21735	13371.14318	HTH-type transcriptional regulator TreR	–
	*ots*A	GM298_03275	196622.198046	Alpha, alpha-trehalose-phosphate synthase	2.4.1.15
	*ots*B	GM298_03270	195844.196647	Trehalose-phosphatase	3.1.3.12
	*lam*B	GM298_21860	6246.7559	Maltoporin LamB	–
**Abiotic stress** **Description**					
Cold Shock protein	*csp*A	GM298_12370	210631.210843	RNA chaperone/antiterminator CspA	–
	*csp*E	GM298_03640	272300.272509	Transcription antiterminator/RNA stability regulator CspE	–
	*csp*D	GM298_04675	137542.137763	Cold shock-like protein CspD	–
**Heat Shock protein**	*smp*B	GM298_07055	306189.306671	SsrA-binding protein SmpB	–
	*ibp*A	GM298_11605	52643.53053	Heat shock chaperone IbpA	–
	*ibp*B	GM298_11610	53190.53618	Heat shock chaperone IbpB	–
	*hsp*Q	GM298_05075	238055.238372	Heat shock protein HspQ	–
	*dna*J	GM298_09430	154155.155300	Molecular chaperone DnaJ	–
	*dna*K	GM298_09435	155388.157301	Molecular chaperone DnaK	–
	*rpo*H	GM298_18075	93268.94125	RNA polymerase sigma factor RpoH	–
	*lep*A	GM298_15045	119042.120841	Elongation factor 4	3.6.5.n1
	*grp*E	GM298_07085	310871.311464	Nucleotide exchange factor GrpE	–
**Drought resistance**	*nha*A	GM298_09425	152809.153984	Na+/H+ antiporter NhaA	–
	*cha*A	GM298_17370	47913.49013	Sodium-potassium/proton antiporter ChaA	–
	*cha*B	GM298_17375	49284.49514	Putative cation transport regulator ChaB	–
	*pro*A	GM298_10450	69705.70958	Glutamate-5-semialdehyde dehydrogenase	1.2.1.41
	*pro*B	GM298_10445	68590.69693	Glutamate 5-kinase	2.7.2.11
	*pro*Q	GM298_03590	263405.264091	RNA chaperone ProQ	–
	*pro*V	GM298_06810	255441.256652	Glycine betaine/L-proline ABC transporter ATP-binding protein ProV	–
	*pro*W	GM298_06805	254393.255457	Glycine betaine/L-proline ABC transporter permease ProW	–
	*pro*X	GM298_06800	253388.254383	Glycine betaine/L-proline ABC transporter substrate-binding protein ProX	–
	*pro*P	GM298_17635	1491.2993	Glycine betaine/L-proline transporter ProP	
	*pro*S	GM298_2152	4993.6711	Proline–tRNA ligase	6.1.1.15
	*bet*A	GM298_16415	95458.97122	Choline dehydrogenase	1.1.99.1
	*bet*B	GM298_16420	97136.98608	Betaine-aldehyde dehydrogenase	1.2.1.8
	*bet*T	GM298_16430	99338.101371	Choline BCCT transporter BetT	
	*trk*A	GM298_22580	7764.9140	Trk system potassium transporter TrkA	
	*trk*H	GM298_19395	80799.82250	Trk system potassium transporter TrkH	–
	*kdp*A	GM298_19955	40526.42205	Potassium-transporting ATPase subunit KdpA	–
	*kdp*B	GM298_19950	38459.40507	Potassium-transporting ATPase subunit Kdp	
	*kdp*C	GM298_19945	37871.38446	Potassium-transporting ATPase subunit KdpC	
	*kdp*F	GM298_19935	34509.35186	Two-component system response regulator KdpE	–

### Pesticide-degrading protein model validation and evaluation

A significant number of genes whose products were responsible for pesticide degradation (*amp*D, *glp*ABQ, *pde*HR, *pep*ABDQ, *phn*FDGHJKLMOP, *paaC*, *hpx*KW, amidohydrolase, and alpha/beta fold hydrolase) were identified in the genome of HSTU-ASh6 (Supplementary Table 2).

For model validation, 39 modeled proteins were analyzed using the SAVESv6.0-structure validation server, among which 28 were validated according to VERIFY (3D-1D score), with their score recorded as 80.05–97.97%. Among these, alpha/beta fold hydrolase family protein (GM298_09990) had the maximum ERRAT quality score of 97.35 (Table 6). The individual protein average TM score, RMSD, IDEN, and Cov were 0.95, 0.75, 0.51, and 0.97, respectively, based on the I-TASSER result (Table 6). Moreover, the Ramachandran plot regions, including the most favored regions (A, B, L), additional allowed regions (a, b, l, p), generously allowed regions (∼, ∼b, ∼l, ∼p), and disallowed regions of the model proteins, are presented in Table 6. Consequently, the 28 validated model proteins were selected for virtual screening with 105 small molecules.

**TABLE 6:** Pesticide degrading model proteins quality assessment of *Enterobacter* sp. HSTU-ASh6 strain.

Model protein	Best PDB hit	TM-score, RMSD, IDEN, Cov	α –helix, β strand, η –coil, disordered	ERRAT (quality score)	VERIFY (3D-1D score)%	Ramachandran plot (core, allow, gener, disallow)
AmpD	1j3gA	0.994, 0.41, 0.809, 1.00	22, 15, 61, 2	83.33	89.62	61.2, 27.6, 9.2, 2.0
GlpA	2qcuB	0.877, 1.00, 0.241, 0.889	35, 19, 44, 4	94.19	78.97	72.5, 18.6, 6.4, 2.5
GlpB	6uziA	0.761, 1.89, 0.145, 0.802	26, 19, 54, 0	64.23	62.47	51.5, 34.8, 8.8, 5.0
GlpQ	1ydyA	0.927, 0.33, 0.857, 0.929	28, 13, 57, 7	85.79	90.93	76.1, 18.7, 3.5, 1.6
PdeH	4rnfA	0.957, 1.25, 0.185, 0.988	39, 18, 41, 6	91.96	74.32	85.0, 12.4, 1.3, 1.3
PdeR	5xgbA	0.810, 0.74, 0.256, 0.815	41, 25, 33, 1	81.52	71.95	72.9, 21.0, 5.1, 1.0
PepA	1gytL	0.999, 0.24, 0.972, 1.000	33, 20, 46, 1	94.30	97.61	85.2, 12.5, 2.1, 0.2
PepB	6cxdA	0.968, 0.27, 0.783, 0.970	34, 20, 45, 1	94.76	93.69	77.7, 18.3, 2.4, 1.6
PepD	3mruA	0.999, 0.27, 0.631, 1.000	26, 25, 48, 3	94.33	95.67	80.5, 16.1, 2.7, 0.7
pepQ	4qr8A	0.995, 0.23, 0.898, 0.996	27, 17, 55, 1	93.07	92.33	80.3, 17.6, 1.0, 1.0
PhnD	3p7iA	0.884, 0.44, 0.897, 0.888	43, 17, 39, 1	84.54	72.22	79.5, 14.2, 4.6, 1.7
PhnF	2wv0D	0.980, 0.51, 0.193, 0.988	28, 32, 39, 4	88.36	83.82	74.3, 21.6, 4.1, 0.0
PhnG	4xb6A	0.941, 0.69, 0.750, 0.960	44, 26, 29, 5	95.03	66.67	85.1, 13.4, 1.5, 0.0
PhnH	2fsuA	0.852, 0.67, 0.850, 0.861	24, 21, 54, 3	89.24	88.66	70.4, 23.7, 5.3, 0.6
PhnJ	4xb6D	0.984, 0.23, 0.957, 0.986	22, 19, 57, 4	85.34	85.05	78.0, 17.8, 3.3, 0.8
PhnK	4fwiB	0.980, 0.80, 0.320, 0.992	37, 23, 39, 2	93.41	90.44	80.2, 16.5, 1.4, 1.9
PhnL	5nikJ	0.929, 1.05, 0.290, 0.960	34, 24, 41, 0	93.57	89.38	78.4, 18.6, 2.0, 1.0
PhnM	1k1dA	0.890, 2.18, 0.169, 0.955	32, 22, 45, 0	78.10	75.93	61.9, 28.7, 5.7, 3.6
PhnO	1s5kA	0.914, 1.93, 0.168, 0.993	35, 33, 31, 2	97.05	80.56	74.6, 19.2, 3.8, 2.3
PhnP	3g1pB	0.989, 0.31, 0.768, 0.992	12, 28, 58, 0	84.23	98.41	70.1, 24.3, 2.3, 3.3
Carboxylesterase	1maaD	0.951, 1.38, 0.292, 0.976	30, 13, 55, 1	80.98	83.63	71.4, 21.4, 3.9, 2.9
PaaC	1otkA	0.978, 0.26, 0.802, 0.980	47, 12, 40, 1	95.83	89.92	91.3, 7.3, 0.90, 0.5
HpxK	5i4mA	0.906, 2.25, 0.321, 0.985	32, 22, 44, 1	88.22	94.65	78.4, 17.3, 2.0, 2.3
HpxW	4y23A	0.954, 1.63, 0.242, 0.981	33, 15, 51, 1	86.12	97.72	73.6, 22.1, 2.8, 1.6
Amidohydrolase (GM298_10355)	2e11A	0.984, 0.72, 0.467, 0.996	25, 32, 42, 1	84.55	87.50	75.9, 21.0, 1.3, 1.8
Amidohydrolase (GM298_00905)	4ewtA	0.989, 0.56, 0.334, 0.995	31, 24, 44, 0	89.04	95.17	78.1, 17.2, 2.8, 1.9
AHFP (GM298_01650)	4l5pA	0.936, 1.04, 0.271, 0.954	43, 11, 45, 0	93.15	90.70	78.2, 17.7, 1.4, 2.7
AHFP (GM298_06905)	2qt3B	0.842, 1.26, 0.213, 0.861	31, 16, 52, 11	89.357	74.51	67.0, 23.6, 6.7, 2.7
AHFP (GM298_20245)	3nqbA	0.954, 0.42, 0.507, 0.956	25, 26, 47, 2	83.90	97.97	75.8, 18.7, 2.9, 2.6
Amidohydrolase (GM298_14085)	4ewtA	0.870, 0.67, 0.254, 0.876	34, 20, 45, 0	85.545	85.78	71.9, 23.5, 3.2, 1.3
Amidohydrolase (GM298_21680)	2icsA	0.935, 0.92, 0.453, 0.950	26, 22, 51, 3	82.92	92.31	75.4, 20.1, 2.4, 2.1
GM298_09975AHFP	3ighX	0.758, 0.78, 0.204, 0.764	28, 16, 54, 10	75.12	88.60	80.5, 15.7, 2.8, 1.0
GM298_00815_ABFH	4lxgA	0.944, 1.60, 0.184, 0.996	36, 17, 45, 1	87.65	94.42	75.7, 19.0, 3.3, 1.9
GM298_08590_ABH	4ypvA	0.983, 0.66, 0.393, 0.993	31, 20, 47, 1	95.89	94.33	80.4, 15.3, 3.9, 0.4
GM298_09990_ABFH	1va4A	0.989, 0.36, 0.742, 0.993	39, 15, 45, 0	97.35	96.70	82.8, 14.6, 2.1, 0.4
GM298_18675_ABH	4ypvA	0.992, 0.58, 0.348, 1.000	38, 16, 45, 1	88.17	89.80	89.0, 9.5, 1.1, 0.4
GM298_01305_ABFH	3fvr	0.579, 2.12, 0.171, 0.607	31, 16, 51, 9	61.64	72.62	55.0, 30.4, 10.4, 4.2

**FIGURE 5:**
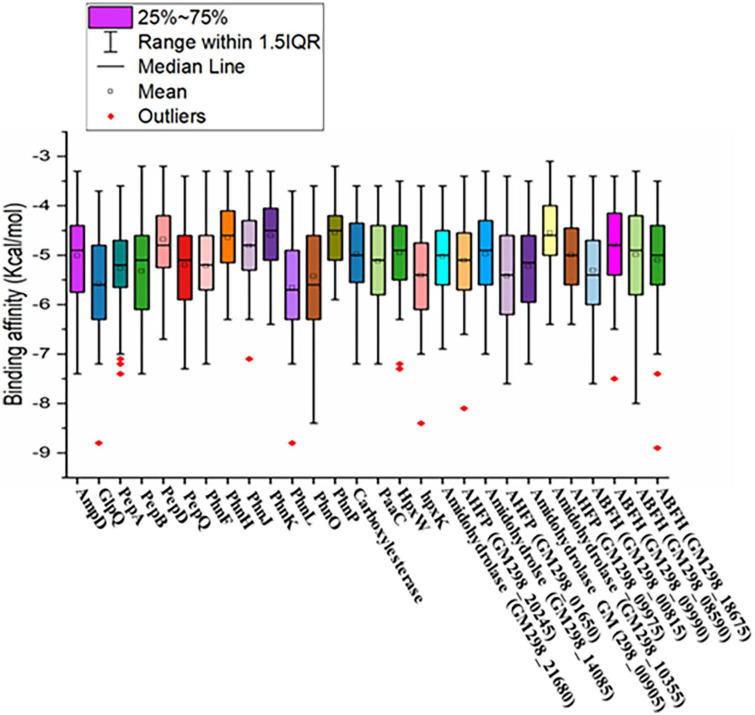
Graphical representation of virtual screening results of pesticide-degrading validated model proteins with 105 different organophosphorus pesticides and other common pesticides applied in fields.

A total of 105 different pesticides, including weed killers and organophosphorus nerve agents, were screened with the 28 validated model proteins of *Enterobacter* sp. HSTU-ASh6 (Figure 5). The score ranged from –8.8 to –3.1 (kcal/mol). The highest negative score was recorded for cypermethrin with phnL (–8.8 kcal/mol), and the lowest negative score was found for demephion-O with amidohydrolase (GM298_10355) (–3.1 kcal/mol). The box plot analysis revealed that each box with the bold line represents median values, and the square sign indicates mean values. Moreover, the upper and lower lines in the box plots represent minimum and maximum values, respectively. Interestingly, most values were found between the 25th and 75th percentile. The 25th and 75th percentile quartile differences indicated the interquartile range. Surprisingly, in this dataset, some outlier data were observed, indicating the presence of some small molecules possessing an excellent binding affinity with respective proteins. It was also observed that the nine proteins’ outlier binding affinities values were outside of IQR1.5.

### Catalytic interactions of model proteins with selective pesticides

**FIGURE 6:**
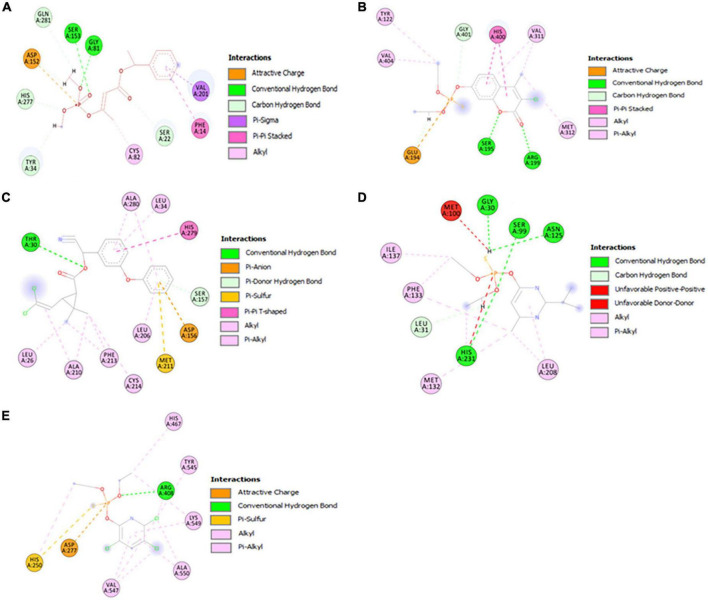
Visualization of the catalytic interactions of potential model proteins with pesticides. **(A)** α/β hydrolase (GM298_18675) with crotoxyphos, **(B)** carboxylesterase with coumaphos, **(C)** alpha/beta fold hydrolase (GM298_08590) with cypermethrin, **(D)** alpha/beta fold hydrolase (GM298_00815) protein with diazinon, **(E)** amidohydrolase family protein (GM298_20245) with chlorpyrifos.

As shown in Figure 6, the alpha/beta fold hydrolase (GM298_18675) of *Enterobacter* sp. HSTU-ASh6 anchored with crotoxyphos and formed a potential catalytic triad in the binding pocket region with residues Ser153-His277-Asp152. Ser153 and Gly81 specifically mediated the attractive charge connection with the +P-atom of crotoxyphos and the typical H-bond contact with the O atom (Figure 6A). Besides other interactions, Val201 and Phe14 directly interacted with the phosphodiester bond of crotoxyphos via π-sigma and π-π stacked interaction. The interaction distances among the residues of the catalytic site were recorded within < 3 Å, except for Asp152, which was 5.36 Å.

Surprisingly, the residues Ser195, His400, and Glu194 constituted a putative catalytic triad in the binding pocket region of the *Enterobacter* sp. HSTU-ASh6 carboxylesterase. Ser195 and Arg199 interacted with the O atom to form the traditional H-bond, and Glu194 interacted with the +P atom to form phosphodiester bonds and the H atom to interact with the carbon-hydrogen bond of coumaphos (Figure 6B). Moreover, the π-π stacked bond was formed by the His400 residue of carboxylesterase with coumaphos ligand, and it directly interacted with the phosphodiester bond of ligand. The interaction distances among the residues of the catalytic site were recorded within <4 Å.

The alpha/beta fold hydrolase (GM298_08590) formed a potential catalytic triad Ser157-His279-Asp156 in the binding pocket region with cypermethrin (Figure 6C). The interaction distances among the catalytic site residues were recorded within <5.25 Å. Furthermore, the alpha/beta fold hydrolase (GM298_00815) protein–diazinon docked complex demonstrated interaction with multiple residues (Figure 6D). In particular, conventional H-bonds were made by Ser99, His231 with the O atom of the diazinon compound. In addition, the amidohydrolase family protein (GM298_20245) anchored with chlorpyrifos and showed a significant interaction (Figure 6E). For instance, Arg408 interacted with the O atom of chlorpyrifos by a conventional hydrogen bond. Moreover, alkyl and pi-alkyl bonds were made by Val547, Arg408, Ala550, Lys549, His250, His467, and Tyr545 with chlorpyrifos, respectively. Interestingly, all of them were observed with two bonds, except His467 and Tyr545. Only one amino acid made the attractive charge and was interconnected with +P-atom. His250 made pi-sulfur and pi-alkyl bond at a time.

### Molecular dynamics simulation

**FIGURE 7:**
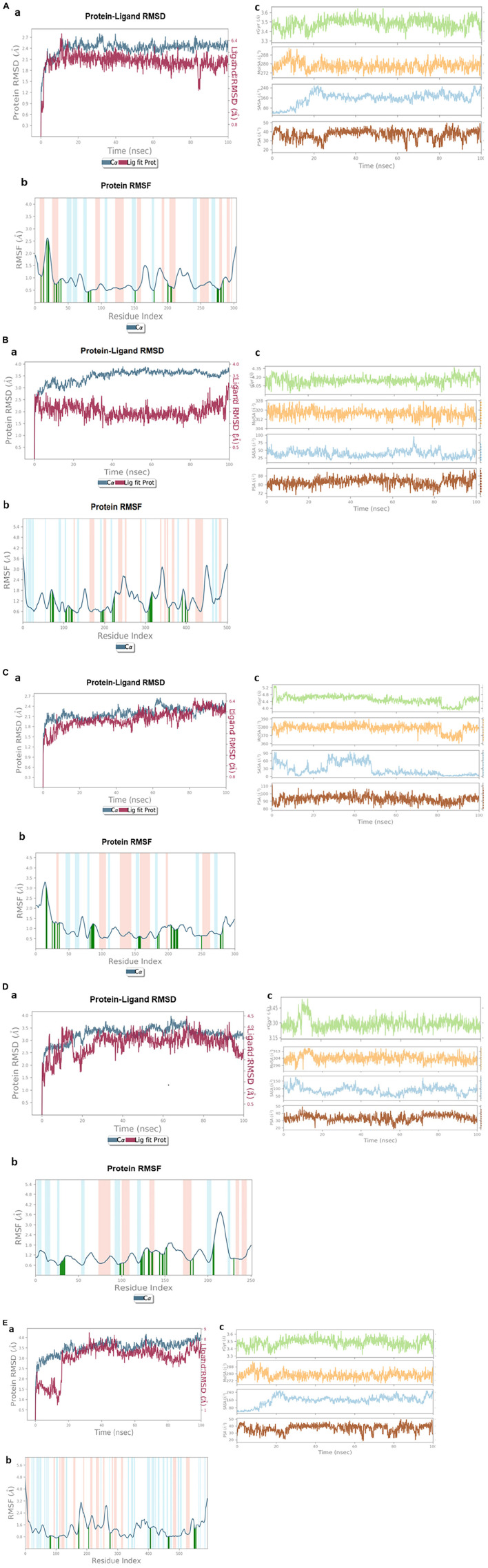
Stability of protein and pesticide interactions. **(A)** Molecular dynamics simulation of α/β hydrolase (GM298_18675)–crotoxyphos complex, (a) protein–ligand RMSD, (b) protein RMSF, and (c) Rg, MolSA, SASA, and PSA. **(B)** Carboxylesterase–coumaphos complex, (a) Protein–ligand RMSD, (b) protein RMSF, and (c) Rg, MolSA, SASA, and PSA. **(C)** α/β hydrolase (GM298_08590)–cypermethrin complex, (a) Protein–ligand RMSD, (b) protein RMSF, and (c) Rg, MolSA, SASA, and PSA. **(D)** α/β hydrolase (GM298_00815)–diazinon complex, (a) Protein–ligand RMSD, (b) protein RMSF, and (c) Rg, MolSA, SASA, and PSA. **(E)** Amidohydrolase (GM298_20245)–chlorpyrifos complex, (a) protein–ligand RMSD, (b) protein RMSF, and (c) Rg, MolSA, SASA, and PSA.

The RMSD plot of the α/β hydrolase (GM298_18675)–crotoxyphos complex was sharply increased from 0 to 4 ns (Figure 7Aa), and it continued up to 30 ns with an RMSD ranging from 2.6 to 3.45 Å. After a slight decrease in the RMSD of the protein, at 35 ns, an increasing parallel trend continued up to 100 ns, with an RMSD ranging from 2.5 to 3.35 Å, except a slight decrease at 83–86 ns of the ligand. In the RMSF plot of the alpha/beta hydrolase (GM298_18675)–crotoxyphos complex (Figure 7Ab), the favorable residues of alpha/beta hydrolase (GM298_18675) with crotoxyphos were observed at Leu10 (0.93 Å), Phe14 (1.161 Å), Lys19 (2.806 Å), Ser21 (3.243 Å), Ser22 (3.639 Å), Arg30 (0.798 Å), Gly33 (0.714 Å), Thr34 (0.636 Å), Ser37 (0.842 Å), Leu40 (0.975 Å), Gly81 (0.43 Å), Cys82 (0.416 Å), Ser85 (0.449 Å), Asp152 (0.378 Å), Tyr181 (0.425 Å), Val201 (0.70 Å), Ile202 (0.659 Å), Thr206 (0.644 Å), Leu207 (0.51 Å), Ile276 (0.542 Å), His277 (0.493 Å), Gly278 (0.456 Å), Gln281 (0.452 Å), Leu282 (0.539 Å), and Ile285 (1.005 Å). The Rg of the α/β hydrolase (GM298_18675)–crotoxyphos complex fluctuated throughout the simulation, with an average value of 3.960 Å. The MolSA, solvent-accessible surface area (SASA), and PSA of the alpha/beta hydrolase (GM298_18675)–crotoxyphos complex insignificantly varied throughout the simulation period. The average MolSA, SASA, and PSA were 310.304, 56.5454, and 81.3709 Å^2^, respectively (Figure 7Ac).

The RMSD plot (Figure 7Ba) of the carboxylesterase–coumaphos complex was sharply increased from 0 to 3 ns, and it continued up to 23 ns, with an RMSD ranging from 2.3 to 3.45 Å. After a slight decrease in the RMSD of the protein, at 35 ns, a parallel increasing trend continued up to 100 ns, with an RMSD ranging from 2.5 to 3.80 Å, except a slight decrease at 83–86 ns of the ligand. In the RMSF plot of the carboxylesterase–coumaphos complex (Figure 7Bb), the favorable residues of carboxylesterase with coumaphos were observed at Glu69 (1.491 Å), Thr73 (1.723 Å), Gly75 (1.948 Å), Gly76 (1.807 Å), Trp106 (0.456 Å), His108 (0.787 Å), Ile114 (0.918 Å), Leu119 (0.923 Å), Pro121 (0.736 Å), Tyr122 (0.66 Å), Phe192 (0.437 Å), Ser195 (0.64 Å), Ala196 (0.829 Å), Arg199 (0.71 Å), Ser221 (0.717 Å), Thr224 (2.112 Å), Leu225 (1.79 Å), Glu308 (0.59 Å), Val311 (0.716 Å), Met312 (0.884 Å), Val314 (1.919 Å), Phe315 (1.881 Å), Ile317 (1.698 Å), Ala359 (0.803 Å), Phe360 (0.643 Å), His391 (1.243 Å), Trp399 (0.976 Å), His400 (0.734 Å), and Val404 (0.761 Å). The Rg of the carboxylesterase–coumaphos complex fluctuated throughout the simulation, with an average value of 4.147 Å. The MolSA, SASA, and PSA of the carboxylesterase–coumaphos complex insignificantly varied throughout the simulation period. The average MolSA, SASA, and PSA were 317.102, 38.885, and 82.488 Å^2^, respectively (Figure 7Bc).

The RMSD plot of the α/β hydrolase (GM298_08590)–cypermethrin complex was sharply increased from 0 to 3 ns (Figure 7Ca), and it continued up to 100 ns with the exception of a few irregularities. The overall RMSD difference ranged from 3.5 to 4.01 Å. In the RMSF plot of the alpha/beta hydrolase (GM298_08590)–cypermethrin complex (Figure 7Cb), the favorable residues of alpha/beta hydrolase (GM298_08590) with cypermethrin were observed at Ala17 (3.112 Å), Pro18 (2.65 Å), Leu26 (0.956 Å), Ala29 (1.522 Å), Thr30 (1.435 Å), Leu34 (1.069 Å), Leu37 (1.135 Å), His83 (0.858 Å), Gly85 (1.275 Å), Gly86 (1.409 Å), Trp87 (1.15 Å), Cys88 (1.027 Å), Leu89 (0.969 Å), Asp156 (0.639 Å), Ser157 (0.688 Å), Ala158 (0.771 Å), Gly159 (0.63 Å), Tyr185 (0.628 Å), Ala187 (0.845 Å), Phe205 (0.93 Å), Leu206 (1.228 Å), Asp209 (0.995 Å), Ala210 (0.974 Å), Met211 (0.878 Å), Phe213 (0.867 Å), Cys214 (0.883 Å), Thr215 (0.929 Å), Leu251 (0.61 Å), His279 (0.561 Å), Ala280 (0.652 Å), and His283 (0.856 Å). The Rg of the alpha/beta hydrolase (GM298_08590)–cypermethrin complex fluctuated throughout the simulation, with an average value of 4.512 Å. The MolSA, SASA, and PSA of the alpha/beta hydrolase (GM298_08590)–cypermethrin complex insignificantly varied throughout the simulation period. The average MolSA, SASA, and PSA were 378.775, 26.194, and 93.0372 Å^2^, respectively (Figure 7Cc).

The RMSD plot of the α/β hydrolase (GM298_00815)–diazinon complex was sharply increased from 0 to 17 ns (Figure 7Da), and it continued up to 75 ns. Next, a slight parallel downward trend appeared from 75 to 100 ns. The overall RMSD difference ranged from 1.2 to 1.7 Å. In the RMSF plot of the alpha/beta fold hydrolase (GM298_00815)–diazinon complex (Figure 7Db), the favorable residues of alpha/beta fold hydrolase (GM298_00815) with diazinon were observed at Gly30 (0.689 Å), Leu31 (0.553 Å), Gly32 (0.767 Å), Cys33 (0.934 Å), Ala34 (0.947 Å), Ala35 (1.37 Å), Ser99 (0.656 Å), Met100 (0.76 Å), Ser103 (0.906 Å), Glu123 (0.66 Å), Pro124 (0.801 Å), Asn125 (1.033 Å), His127 (1.774 Å), Met132 (1.439 Å), Phe 133 (1.301 Å), Ser136 (1.316 Å), Ile137 (0.994 Å), Phe145 (1.217 Å), Gln148 (1.277 Å), Gly149 (1.105 Å), Asp151 (1.429 Å), Met153 (1.143 Å), Val181 (0.822Å), Val184 (0.792 Å), Ser207 (1.098 Å), Leu208 (1.684 Å), and His231 (1.028 Å). The Rg of the alpha/beta fold hydrolase (GM298_00815)–diazinon complex fluctuated throughout the simulation, with an average value of 3.29 Å. The MolSA, SASA, and PSA of the alpha/beta fold hydrolase (GM298_00815)–diazinon complex insignificantly varied throughout the simulation period. The average MolSA, SASA, and PSA were 304.144, 88.6872, and 33.733 Å^2^, respectively (Figure 7Dc).

The RMSD plot of the amidohydrolase (GM298_20245)–chlorpyrifos complex (Figure 7Ea) showed an increasing trend for the ligand protein from 0 to 14 ns. After 17 ns, an increasing parallel trend continued up to 44 ns, with an RMSD ranging from 2.5 to 3.5 Å. Next, a steady and stable RMSD remained up to 80 ns. Then, the RMSD difference was below ∼1.2 Å up to 93 ns, which indicated the stable protein–ligand complex. In the RMSF plot of the amidohydrolase–chlorpyrifos complex (Figure 7Eb), the favorable residues of amidohydrolase with chlorpyrifos were observed at His81 (0.826 Å), Ser84 (0.99 Å), His109 (0.872 Å), Glu110 (0.888 Å), Glu174 (0.906 Å), Met176 (1.848 Å), Arg207 (1.4 Å), Asp276 (1.373 Å), Asp277 (1.118 Å), Ala407 (1.276 Å), Arg408 (1.631 Å), Tyr465 (0.683 Å), Ser466 (1.006 Å), His467 (1.215 Å), Asp468 (0.95 Å), Lys549 (1.393 Å), Ala550 (1.131 Å), Glu552 (1.336 Å), Gly553 (1.338 Å), and Leu556 (1.327 Å). The Rg of the amidohydrolase–chlorpyrifos complex fluctuated throughout the simulation, with an average value of 3.48 Å. The MolSA, SASA, and PSA of the amidohydrolase–chlorpyrifos complex insignificantly varied throughout the simulation period. The average MolSA, SASA, and PSA were 278.25, 156.69, and 37.03 Å^2^, respectively (Figure 7Ec).

## Discussion

Endophytes play an important role in the agricultural sector by enhancing crop production, pathogen resistance, and pesticide detoxification. These characteristic endophytic bacteria can be used as biofertilizers for sustainable agriculture. In the present study, we extensively investigated a newly isolated rice root endophyte strain that might have mineralized chlorpyrifos as its carbon source and demonstrated the tomato PGP activities at germination, vegetative, and reproductive stages. In particular, the strain classification revealed that it newly evolved in Bangladesh and deviated far away from its nearest homologs.

The gram-negative bacterium HSTU-ASh6 showed positive results in the VP test, indicating its capability to produce acetoin in the growth media. It also showed a positive result in the catalase test. Several studies reported that endophytic strain showed catalase positive test (Son et al., 2006; Das et al., 2022). Catalase helps bacteria avoid cellular toxicity. The positive triple sugar iron test indicated that the isolate could ferment three sugars, including lactose, sucrose, and glucose, and iron. As the strain produced gas and exhibited motility properties, it was confirmed to be motility indole urease (MIU)–positive. The strain was also urease-positive, which is important for endophytic bacteria. Urease is an enzyme that splits urea into simple forms of nitrogen that the plants can readily absorb to promote growth. A previous study also demonstrated the occurrence and distribution of urease-positive endophytic bacteria in a legume community (Kumaresan and Suryanarayanan, 2001).

Previous reports have shown that several endophytic bacteria, including *Pseudomonas*, *Serratia*, and *Bacillus* species, can synthesize IAA that is involved in plant root regulation and growth control (Sgroy et al., 2009; Liu et al., 2010). Moreover, endophytes such as *Pantoea*, *Pseudomonas*, *and Serratia* have the ability to fix nitrogen in some plants (Loiret et al., 2004; Das et al., 2022). The present study assumed that the strain HSTU-ASh6 could fix nitrogen in Jensen’s medium. Phosphate-solubilizing bacteria can dissolve insoluble phosphate and increase soil fertility and plant growth (Loiret et al., 2004; Das et al., 2022), which was also demonstrated by the strain HSTU-ASh6. Moreover, the strain enhanced the germination rate and increased tomato plant growth at vegetative and reproductive stages when using 70 and 30% reduced doses of urea. These results indicated that the strain could fix nitrogen in the tomato plant and assist its growth by providing phosphate solubilization, IAA, and ACC deaminase activities. In particular, a massive growth of tomato plants was observed in 100% urea + HSTU-ASh6 strain treatment compared with the control. The multi-branched bushy structure of bacterium-treated tomato plants was due to the secretion of the auxin (Supplementary Figures 3A,B), which is agreed to the Khan et al. (2016). Based on these results, it was confirmed that the bacterium exerted robust growth-promoting effects on tomato plants.

HSTU-ASh6 completely degraded 1 g L^–1^ of chlorpyrifos within 14 days of incubation in the minimal salt medium. A previous study reported that *Enterobacter* sp. B-14 rapidly degraded chlorpyrifos in MSM, and this strain used chlorpyrifos as the only source of carbon and phosphorus (Singh et al., 2004). Das et al. (2022) reported that HSTU strains degraded 42–100% of chlorpyrifos in broth media. Li et al. (2007) showed that *Sphingomonas* sp. DSP2 degraded 100% of chlorpyrifos (100 mg L^–1^) in 24 h. It has also been shown that chlorpyrifos degradation results in the generation of several byproducts such as chlorpyrifos oxon, 3,5,6-trichloro-2-methoxypyridine, and 2-chloro-6-hydroxypyridine (Singh et al., 2004). A significant number of spinoff products were observed, for instance, chlorodihydro-2-pyridone, dihydroxy pyridine, tetrahydro-2-pyridone, maleamide semialdehyde, maleamic acid, and pyruvic acid. It was reported that chlorpyrifos and TCP can be broken down to produce 1,3-bis (1,1-dimethylethyl) and benzene (Abraham and Silambarasan, 2013). Chlorpyrifos oxon, TCP, and DEMP are the metabolic byproducts generated by the breakdown of chlorpyrifos organophosphorus insecticide (Lovecka et al., 2015). In this study, the products generated after chlorpyrifos degradation were phorate sulfoxide, phorate sulfone, chlorpyrifos methyl, TCP, carbophenothion sulfoxide, oxydisulfoton, carbonochloridic acid, 2,4,5-trichlorophenyl ester, thionodemeton sulfone, 3-(2-thienyl)-DL-alanine, chlorpyrifos oxon, diethyl methanephosphonate, which were confirmed through GC–MS/MS analysis (Table 3). To the best of our knowledge, this is the first report describing that diethyl methanephosphonate (DEMP) is a chlorpyrifos metabolic byproduct.

The quality of the genome sequence was predicted according to the GC% of strain DNA. A good-quality sequence is ensured by its GC%, and at least 40–70% of GC% is accepted in research (Abdullah-Al-Mamun et al., 2022). The strain investigated in this study exhibited 55.1% GC% after fast QC analysis, and the sequence quality was confirmed to be excellent.

According to the phylogenetic tree analysis of 16S rRNA genes, *Enterobacter* sp. HSTU-ASh6 was placed with *E. sichuanensis* WCHECL1597 (MG832788) in the sister taxa. They were 45% similar to each other and identified as *E. sichuanensis*. However, depending only on 16S rRNA and the polyphyletic nature of the genus *Enterobacter* make its identification and categorization extremely difficult. Therefore, we further confirmed our result using three housekeeping genes (*recA*, *gyrB*, and *rpoB*), and a complete genome phylogenetic tree was constructed and analyzed separately. The constructed tree revealed that HSTU-ASh6 was appeared as *E. cloacae* A1137. Based on 16S rRNA, *Enterobacter* sp. HSTU-ASh6 was specified as *E. sichuanensis.* However, after the analysis of another three housekeeping genes and complete genome phylogenetic tree analyses, it was identified as *E. cloacae.* However, in the case of *rec*A tree, both *E. cloacae* A1137 and *E. sichuanensis* WCHECL1597 were placed in a different clade with *Enterobacter* sp. HSTU-ASh6, where *E. cloacae* A1137 was located in the first node. According to GGDC web server, a DDH value of >70% indicates that the strain belongs to the same species and >79% based on the formula: 2 indicates that the strain belongs to the same subspecies. In another analysis, an ANI cut-off of > 96% was observed for species declaration. Consequently, our study results suggested that the strain HSTU-ASh6 belonged to *Enterobacter* species because both DDH and ANI values exceeded the cut-off. After progressive Mauve and pangenomic analysis, it was concluded that *Enterobacter* sp. HSTU-ASh6 was completely diversified from its closest strains, which indicates its evolutionary properties. Therefore, *Enterobacter* sp. HSTU-ASh6 might be a new member of the *Enterobacter* species.

*Escherichia coli* and closely related *Enterobacteria* possess both Isc and Suf systems, encoded by the *isc*RSUA-hscBA-fdx-iscX operon and sufABCDSE operon, respectively (Ayala-Castro et al., 2008). In general, the endophytic bacterial genome contains *nif* and *fix* gene clusters for nitrogen fixation. However, a few endophytic bacteria contain other types of gene clusters, namely, Isc and Suf systems, which are responsible for nitrogen fixation in critical situations (Ayala-Castro et al., 2008). The present study showed that the genome of *Enterobacter* sp. HSTU-ASh6 harbors both Isc and Suf systems for biological nitrogen fixation, and the genome machinery also indicated that both Isc and Suf operons were present. To summarize, the PGP mechanism is encoded by genes that are directly linked to the generation of IAA and ACC deaminase and siderophore in *Enterobacter* sp. HSTU-ASh6. In the same way, genes like *nha*A, *cha*AB, *pro*ABpqvwsx, *bet*ABT, *trk*AH, *kdp*ABCEF, and *kdb*D that help the plant deal with drought stress have been found in the genomes, indicating their involvement in drought tolerance by the plant (Das et al., 2022).

The conserved pentapeptide motif G-X-S-X-G is commonly observed in bacterial genome sequences (Schloss and Handelsman, 2003). The genome of *Enterobacter* sp. HSTU-ASh6 encodes proteins such as alpha/beta fold hydrolase (GM298_18675), alpha/beta fold hydrolase (GM298_08590), alpha/beta fold hydrolase (GM298_00815), and carboxylesterase sequences carrying the conserved pentapeptide G-X-S-X-G motif. The hydrolase-encoding genes, for instance, *opd* (McDaniel et al., 1988), *mpd* (Lu et al., 2013), and *ophc*2 (Ningfeng et al., 2004; Shen et al., 2010), are also crucial for the breakdown of pesticides. Das et al. (2022) reported that carboxylesterase and phosphotriesterase are directly involved in the breakdown of organophosphorus insecticides. Haque et al. (2018, 2020) also reported that several *opd* genes (*opd*A, *opd*E, and *opd*D) can degrade a range of organophosphorus insecticides. These results suggested that the genome of *Enterobacter* sp. HSTU-ASh6 retains pesticide-mineralizing genes. In addition, several hydrolases, esterase, and some hypothetical proteins may have catalytic interactions with organophosphorus insecticides, which is beyond the scope of this study.

The molecular docking investigations of pesticide-degrading enzymes with pesticides were subjected with a validity score. The RMSD of model protein indicates its acceptance when compared to a typical protein model. Since a smaller RMSD suggests fewer errors, it is always preferred. The 28 modeled proteins’ average RMSD was 1.0, indicating that the analyses were valid. According to reports, protein models with RMSDs between 0.35 and 2.36 and their nearby homologs are acceptable (Dadheech et al., 2019). Depending on the molecular docking analysis, all five proteins (alpha/beta fold hydrolase (GM298_18675), alpha/beta fold hydrolase (GM298_08590), alpha/beta fold hydrolase (GM298_00815), amidohydrolase family protein (GM298_20245), and carboxyl esterase) with important amino acid residues for catalysis were found within 1.5–5.5 Å, which suggested its involvement in the degradation of organophosphate pesticides. Haque et al. (2018) reported that carboxylesterase provides Ser-His-Glu catalytic triad. The present study indicated that carboxylesterase docked with coumaphos and provided a similar type of Ser-His-Glu catalytic triad. Consequently, the alpha/beta fold hydrolase (GM298_18675) of *Enterobacter* sp. HSTU-ASh6 originated as a potential catalytic triad Ser-His-Asp (Hosokawa, 2008). Therefore, the α/β fold hydrolase (GM298_08590) was docked with cypermethrin and provided Ser-His-Asp catalytic triad (Hosokawa, 2008). Kubiak et al. (2001) detected the Arg-Asp-His catalytic triad in the enzymatic cleavage of the phosphodiester bond. A similar triad (Arg-Asp-His) was observed in the present study, where amidohydrolase docked with chlorpyrifos insecticide. A recent study indicated that the Ser-His-Glu catalytic triad is predominant in long PepEs, and the Ser-His-Asn “catalytic triad” is predominant in short PepEs (Yadav et al., 2019). Interestingly, alpha/beta fold hydrolase (GM298_00815) bonded with diazinon pesticide and formed a new possible catalytic triad Ser-His-Asn.

Five different proteins with five different pesticides complex were subjected to MD simulation for 100 ns. The stability of the protein-ligand complex was determined (Patel et al., 2021) by comparing the RMSD and RMSF values of the unbound protein structure. In MD simulations, the RMSD parameter is used to evaluate the coherence and flexibility of proteins as well as to keep track of the separation between their atoms and backbones (Sargsyan et al., 2017). While a greater RMSD value signifies relatively less stability of the protein-ligand complex, a lower RMSD value throughout the simulation shows higher stability of the protein-ligand complex. It was shown that the enzyme-substrate complexes are stable for the biodegradation of pesticides by a slight fluctuation with lower smaller RMSD (Lee et al., 2021). The results showed that the MD simulations were stable at 100 ns for the insecticides chlorpyrifos, diazinon, crotoxyphos, cypermethrin, and coumaphos complex with pesticides degrading potential proteins (Figures 7A–E). The RMSF number reflects how each protein’s amino acid moved and changed throughout the simulation. More flexibility during the simulation is implied by higher RMSF values, whilst superior stability is indicated by lower RMSF values. In this study, the RMSF values were different for the chlorpyrifos–amidohydrolase protein family (GM298_20245), diazinon–α/β fold hydrolase (GM298_00815), crotoxyphos–α/β fold hydrolase (GM298_18675), cypermethrin–alpha/beta fold hydrolase (GM298_08590), and coumaphos–carboxylesterase complexes, suggesting that the RMSF of these complexes was stable during the catalytic reactions. Previously, researchers have used RMSF to examine enzyme-pesticide complexes (Lee et al., 2021). The microbial enzymes are responsible for the biodegradation of pollutants like pesticides and they are interconnected (Joshi et al., 2021; Bhatt and Maheshwari, 2022). The changes in compactness of an enzyme-substrate complex are described using the radius of gyration (Rg). It refers to the folding and unfolding of proteins during MD simulations. Enzyme folding indicates Rg stability, whereas fluctuations in Rg signify enzyme unfolding (Adams et al., 2008). The results of Rg and PSA revealed that every enzyme-substrate complex displayed stable compactness in five instances of protein-ligand complexes, indicating that these are superiorly superimposed on each other and perfectly overlaid. SASA stands for solvent-assisted structure-activity relationship. It foresees the structural alterations that take place during interactions. All five protein-ligand complexes’ SASA values during the 100-ns MD simulations of the enzyme-substrate complexes were significantly stable, indicating that the protein structure remained unchanged.

## Conclusion

The endophytic and pesticide-degrading strain *Enterobacter* sp. HSTU-ASh6 isolated from rice roots significantly affected plant growth and pesticide detoxification. Housekeeping gene and whole genome phylogenetic tree, ANI, and DDH genomic analysis confirmed that *Enterobacter* sp. HSTU-ASh6 is a new species of *Enterobacter* strain. Whole genome sequencing also confirmed the presence of genes involved in plant growth, stress tolerance, and pesticide degradation. Therefore, the utilization of this remarkably versatile PGPB may be an essential eco-friendly alternative to improve crop growth and pesticide detoxification.

## Nucleotide sequence accession number

The whole Genome sequence of the *Enterobacter* sp. HSTU-ASh6 strains was deposited at NCBI GenBank under the BioProject PRJNA591446, BioSample SAMN13387920 and accession number: WSPD00000000. In addition, the 16S rRNA gene sequence of the strain was deposited in the NCBI with accession number: MZ021583.

## Data availability statement

The datasets presented in this study can be found in online repositories. The names of the repository/repositories and accession number(s) can be found below: BioProject: PRJNA591446, BioSample: SAMN13387920, and accession number: WSPD00000000.

## Author contributions

MAH: conceptualization, private funding, experimentation, data analysis, and write the manuscript. MSH: experimentation, data analysis, and write first draft of the manuscript. MA: analyze GC–MS/MS. IA and HP: conduct MD simulation. AR: critical revision and partial writing. KC: conceptualization, resource, critical reviewing, editing, proofreading, and fund acquisition. All authors contributed to the article and approved the submitted version.

## Footnotes

1
http://darlinglab.org/mauve/mauve.html

2
https://saves.mbi.ucla.edu/

3
https://pubchem.ncbi.nlm.nih.gov/
