# Supplementary material for: Unveiling chlorpyrifos mineralizing and tomato plant-growth activities of Enterobacter sp. strain HSTU-ASh6 using biochemical tests, field experiments, genomics, and in silico analyses
Source: Front Microbiol. 2022 Nov 29;13:1060554. doi: 10.3389/fmicb.2022.1060554 (PMC9745158; doi:10.3389/fmicb.2022.1060554)
Supplement: Supplementary file 1 [file Data_Sheet_1.docx]

Table S1: Average Nucleotide Identity (ANI) (%) based on whole genome alignments

|  | ***Enterobacter* sp. HSTU-ASh6** | ***Enterobacter asburiae* L1** | ***Enterobacter asburiae* strain ATCC 35953** | ***Enterobacter cloacae* complex sp. ECNIH7** | ***Enterobacter sp.* Crenshaw** | ***Enterobacter cloacae* strain AR 0073** | ***Enterobacter bugandensis* strain 220** | ***Enterobacter kobei* strain C16** | ***Enterobacter chengduensis* strain WCHECl-C4** | ***Enterobacter asburiae* str. AEB30** | ***Enterobacter roggenkampii* strain Ecl 20 981** | ***Enterobacter asburiae* strain RHBSTW-01009** | ***Enterobacter roggenkampii* strain RHBSTW-00695** | ***Enterobacter cloacae* strain A1137** | ***Enterobacter sichuanensis* strain SGAir0282** |
| --- | --- | --- | --- | --- | --- | --- | --- | --- | --- | --- | --- | --- | --- | --- | --- |
| ***Enterobacter* sp*.* HSTU-ASh6** | ***** | **90.82** | **90.99** | **90.69** | **90.85** | **91.03** | **89.32** | **89.01** | **90.19** | **90.91** | **91.06** | **91.02** | **91.12** | **98.89** | **98.12** |
| ***Enterobacter asburiae* L1** | **91.07** | ***** | **98.53** | **96.70** | **96.85** | **92.91** | **91.22** | **90.27** | **92.90** | **94.31** | **92.83** | **98.26** | **92.71** | **91.15** | **91.09** |
| ***Enterobacter asburiae* strain ATCC 35953** | **91.26** | **98.60** | ***** | **96.63** | **96.90** | **92.90** | **91.40** | **90.53** | **92.92** | **94.38** | **92.89** | **99.46** | **92.85** | **91.32** | **91.35** |
| ***Enterobacter cloacae* complex *sp.* ECNIH7** | **90.62** | **96.31** | **96.30** | ***** | **96.18** | **92.80** | **90.71** | **90.09** | **92.71** | **93.74** | **93.06** | **96.19** | **92.47** | **90.86** | **90.72** |
| ***Enterobacter sp. Crenshaw*** | **91.19** | **96.80** | **96.86** | **96.61** | ***** | **92.85** | **91.26** | **90.33** | **92.88** | **94.17** | **92.81** | **96.73** | **92.74** | **91.12** | **91.22** |
| ***Enterobacter cloacae* strain AR 0073** | **91.28** | **92.83** | **92.85** | **92.87** | **92.70** | ***** | **91.02** | **90.09** | **92.19** | **93.73** | **98.14** | **92.92** | **98.17** | **91.58** | **91.23** |
| ***Enterobacter bugandensis* strain 220** | **89.46** | **91.13** | **91.18** | **90.97** | **91.11** | **90.97** | ***** | **90.50** | **90.38** | **90.83** | **91.00** | **91.21** | **90.88** | **89.52** | **89.49** |
| ***Enterobacter kobei* strain C16** | **89.29** | **90.17** | **90.28** | **90.23** | **90.18** | **90.12** | **90.61** | ***** | **90.60** | **90.00** | **90.17** | **90.56** | **89.80** | **89.40** | **89.22** |
| ***Enterobacter chengduensis* strain WCHECl-C4** | **90.37** | **92.77** | **92.79** | **92.71** | **92.64** | **92.11** | **90.42** | **90.69** | ***** | **92.58** | **92.39** | **92.91** | **92.01** | **90.53** | **90.34** |
| ***Enterobacter asburiae* str. AEB30** | **91.25** | **94.30** | **94.40** | **94.14** | **94.20** | **93.82** | **90.97** | **90.09** | **92.75** | ***** | **93.68** | **94.27** | **93.72** | **91.22** | **91.29** |
| ***Enterobacter roggenkampii* strain Ecl 20 981** | **91.31** | **92.71** | **92.58** | **93.06** | **92.60** | **98.01** | **91.02** | **90.21** | **92.54** | **93.54** | ***** | **92.81** | **97.92** | **91.37** | **91.30** |
| ***Enterobacter asburiae* strain RHBSTW-01009** | **91.30** | **98.45** | **99.41** | **96.74** | **96.85** | **93.17** | **91.41** | **90.78** | **93.17** | **94.28** | **93.10** | ***** | **92.98** | **91.63** | **91.37** |
| ***Enterobacter roggenkampii* strain RHBSTW-00695** | **91.41** | **92.80** | **92.89** | **92.93** | **92.77** | **98.40** | **90.98** | **89.96** | **92.17** | **93.71** | **98.27** | **92.86** | ***** | **91.53** | **91.39** |
| ***Enterobacter cloacae* strain A1137** | **98.71** | **90.71** | **90.87** | **90.54** | **90.63** | **91.09** | **89.22** | **89.08** | **90.18** | **90.75** | **91.02** | **91.16** | **91.01** | ***** | **97.89** |
| ***Enterobacter sichuanensis* strain SGAir0282** | **98.06** | **90.88** | **91.11** | **90.80** | **90.90** | **91.07** | **89.34** | **88.93** | **90.19** | **90.99** | **91.12** | **91.03** | **91.05** | **98.03** | ***** |

Table S2: Pesticide degrading associated genes of *Enterobacter* sp. HSTU-ASh6

| **Gene Name** | **Locus Tag** | **CDS** | **Product** | **E.C. number** |
| --- | --- | --- | --- | --- |
| *amp*D | GM298_09055 | 65656..66207 | 1,6-anhydro-N-acetylmuramyl-L-alanine amidase  AmpD | 3.5.1.28 |
| *glp*A | GM298_12875 | 87250..88878 | anaerobic glycerol-3-phosphate dehydrogenase subunit A | 1.1.5.3 |
| *glp*B | GM298_12880 | 88868..90085 | glycerol-3-phosphate dehydrogenase subunit GlpB | 1.1.5.3 |
| *glp*Q | GM298_12865 | 84569..85630 | glycerophosphodiester phosphodiesterase | 3.1.4.46 |
| *pde*H | GM298_22520 | 23992..24765 | cyclic-guanylate-specific phosphodiesterase | 3.1.4.52 |
| *pde*R | GM298_00335 | 59033..61024 | cyclic di-GMP phosphodiesterase | 3.1.4.52 |
| *pep*A | GM298_07505 | 69163..70674 | leucyl aminopeptidase | 3.4.11.1 |
| *pep*B | GM298_14865 | 83158..84444 | aminopeptidase PepB | 3.4.11.23 |
| *pep*D | GM298_10420 | 63156..64613 | cytosol nonspecific dipeptidase | 3.4.13.18 |
| *pep*Q | GM298_19385 | 78814..80145 | Xaa-Pro dipeptidase | 3.4.13.9 |
| *phn*F | GM298_17680 | 12308..13033 | phosphonate metabolism transcriptional regulator PhnF | - |
| *phn*D | GM298_17670 | 10409..11425 | phosphonate ABC transporter substrate-binding protein | - |
| *phn*G | GM298_17685 | 13034..13486 | phosphonate C-P lyase system protein PhnG | - |
| *phn*H | GM298_17690 | 13483..14067 | phosphonate C-P lyase system protein PhnH | 2.7.8.37 |
| *phn*J | GM298_17700 | 15124..15969 | alpha-D-ribose 1-methylphosphonate 5-phosphate C-P-lyase PhnJ | - |
| *phn*K | GM298_17705 | 15966..16721 | phosphonate C-P lyase system protein PhnK | - |
| *phn*L | GM298_17710 | 16807..17487 | phosphonate C-P lyase system protein PhnL | - |
| *phn*M | GM298_17715 | 17484..18620 | alpha-D-ribose 1-methylphosphonate 5-triphosphate diphosphatase | 3.6.1.63 |
| *phn*O | GM298_17725 | 19161..19595 | aminoalkylphosphonate N-acetyltransferase | 2.3.1.- |
| *phn*P | GM298_17730 | 19605..20363 | phosphonate metabolism protein PhnP | 3.1.4.55 |
| *-* | GM298_13095 | 136090..136641 | Phosphodiesterase | 3.1.4.- |
| *paa*C | GM298_20895 | 7936..9360 | 3-hydroxyacyl-CoA dehydrogenase PaaC | - |
| *hpx*K | GM298_05470 | 311191..312426 | allantoate amidohydrolase | - |
| *hpx*W | GM298_05435 | 304356..305939 | oxamate amidohydrolase | - |
| - | GM298_10355 | 49490..50260 | Amidohydrolase | - |
| - | GM298_00905 | 192279..193400 | Amidohydrolase | - |
| - | GM298_01650 | 350669..351703 | amidohydrolase family protein | - |
| - | GM298_06905 | 272079..273458 | amidohydrolase family protein | - |
| - | GM298_20245 | 35068..36846 | amidohydrolase family protein | - |
| - | GM298_14085 | 96207..97517 | Amidohydrolse | - |
| - | GM298_21680 | 389..1522 | Amidohydrolase | - |
| - | GM298_02010 | 419076..420581 | carboxylesterase family protein |  |
| - | GM298_00815 | 170389..171144 | alpha/beta fold hydrolase | - |
| - | GM298_09990 | 265643..266464 | alpha/beta fold hydrolase | - |
| - | GM298_08590 | 284169..285071 | alpha/beta hydrolase fold domain-containing protein | - |
| - | GM298_18675 | 20691..21608 | alpha/beta hydrolase fold domain-containing protein | - |

**Table S3: Virtual screening of validated model proteins with 105 organophosphorus pesticides**

| **Ligand Name** | **AmpD** | **GlpQ** | **PepA** | **PepB** | **PepD** | **pepQ** | **PhnF** | **PhnH** | **PhnJ** | **PhnK** | **PhnL** | **PhnO** | **PhnP** | **Carboxylesterase** | **PaaC** | **hpxW** | **hpxK** | **Amidohydrolase (GM298_21680)** | **AHFP (GM298_20245)** | **Amidohydrolse (GM298_14085)** | **AHFP (GM298_01650)** | **Amidohydrolase**  **GM(298_00905)** | **Amidohydrolase (GM298_10355)** | **AHFP (GM298_09975)** | **ABFH (GM298_00815)** | **ABFH (GM298_09990)** | **ABFH (GM298_08590)** | **ABFH (GM298_18675)** |  |
| --- | --- | --- | --- | --- | --- | --- | --- | --- | --- | --- | --- | --- | --- | --- | --- | --- | --- | --- | --- | --- | --- | --- | --- | --- | --- | --- | --- | --- | --- |
| **Acephate** | -4.4 | -4.7 | -4.4 | -4.7 | -3.8 | -4.8 | -4.5 | -3.9 | -3.9 | -3.6 | -4.4 | -4.3 | -3.8 | -4.2 | -4.2 | -4.6 | -4.4 | -4.4 | -4.2 | -4.3 | -4.2 | -4.5 | -3.6 | -4.4 | -4 | -4 | -4.1 | -4 |  |
| **Azamethiphos** | -5.5 | -6 | -5.2 | -7.3 | -5.3 | -5.7 | -6.1 | -5.1 | -4.9 | -5 | -6.4 | -5.8 | -4.6 | -5.4 | -6 | -5.5 | -6 | -5.8 | -4.7 | -6.4 | -5.4 | -5.5 | -4.8 | -5.4 | -6.1 | -4.5 | -5.4 | -5.9 |  |
| **Azinphos_ethyl** | -6.4 | -6.6 | -7.1 | -7.1 | -5.3 | -6.4 | -6.6 | -5.2 | -5.6 | -5.7 | -6.6 | -7 | -5 | -6 | -6 | -5.6 | -6.3 | -6.2 | -6.2 | -6.3 | -7.3 | -7 | -5.8 | -6.4 | -6.1 | -5.8 | -6 | -5.5 |  |
| **Azinphos_methyl** | -6.7 | -6.8 | -7.4 | -7.1 | -5.3 | -6.7 | -6.8 | -5.1 | -5.8 | -5.7 | -6.7 | -7 | -5.1 | -5.9 | -6.6 | -5.9 | -6.2 | -6 | -6.5 | -6.4 | -7.2 | -7.2 | -5.8 | -6.4 | -6.7 | -5.5 | -5.8 | -5.8 |  |
| **Bromophos_ethyl** | -4.9 | -5.7 | -4.7 | -4.4 | -4.4 | -4.5 | -5.4 | -4.7 | -4.6 | -4.2 | -5.6 | -5.6 | -4.9 | -5.2 | -5 | -4.6 | -5.3 | -4.7 | -4.3 | -5.8 | -6.3 | -4.9 | -4.3 | -4.6 | -5.5 | -4.8 | -5.1 | -4.8 |  |
| **Bromophos** | -4.8 | -5.4 | -4.7 | -4.7 | -4.4 | -4.7 | -5.2 | -4.4 | -4.5 | -4.5 | -5.7 | -5.6 | -4.6 | -5 | -5.1 | -4.9 | -5.2 | -5 | -4.5 | -4.8 | -5.6 | -4.8 | -4.5 | -4.8 | -5.2 | -4.4 | -5 | -4.7 |  |
| **Cadusafos** | -4.4 | -4.9 | -4.5 | -4.3 | -4.3 | -4.3 | -4.8 | -4 | -4.2 | -4 | -5.2 | -5.1 | -4.3 | -4.4 | -4.1 | -4.1 | -5.1 | -4.5 | -4.5 | -4.4 | -4.3 | -4.9 | -4 | -4.5 | -4.9 | -4.7 | -4.6 | -4.8 |  |
| **Carbendazim** | -6.6 | -6.3 | -6.7 | -7.2 | -5.4 | -6.5 | -6.4 | -5.6 | -5.7 | -5.5 | -7.1 | -6.9 | -3.9 | -5.8 | -6.8 | -6.3 | -6.1 | -6.1 | -5.8 | -6.6 | -6.4 | -6.1 | -6.4 | -5.9 | -6 | -5.7 | -5.7 | -6.3 |  |
| **Carbofuran** | -5.9 | -6.6 | -6.1 | -7.4 | -5.7 | -6.2 | -7 | -5.2 | -5.9 | -6 | -7.2 | -6.8 | -5.8 | -6.4 | -6.8 | -6.1 | -6.6 | -6 | -6.1 | -6.2 | -5.9 | -6.2 | -5.4 | -5.9 | -6.8 | -6.5 | -6.1 | -6.6 |  |
| **Chlormephos** | -3.5 | -4.2 | -3.9 | -3.7 | -3.3 | -3.6 | -4 | -3.3 | -3.6 | -3.6 | -4.4 | -3.8 | -5.6 | -3.6 | -3.6 | -4.3 | -4.1 | -3.8 | -3.7 | -3.5 | -4.1 | -3.9 | -5.3 | -3.8 | -3.8 | -3.7 | -3.5 | -3.9 |  |
| **Chlorphoxim** | -6.4 | -6.8 | -6 | -6.6 | -5.3 | -6.1 | -6.5 | -5.5 | -6 | -5.2 | -6.7 | -6.3 | -3.2 | -5.9 | -6.1 | -5.7 | -6.2 | -5.8 | -6.2 | -5.6 | -6.9 | -6.2 | -5.3 | -5.6 | -6.3 | -5.6 | -6 | -6.8 |  |
| **Chlorpyrifos** | -5 | -5.6 | -5.1 | -5.2 | -4.9 | -5.2 | -5.4 | -4.8 | -4.9 | -4.7 | -5.9 | -5.7 | -5.1 | -5.5 | -5 | -5.1 | -5.7 | -5.1 | -5.3 | -5.1 | -6.4 | -5 | -4.7 | -5.1 | -5.5 | -4.7 | -5.2 | -5.4 |  |
| **Chlorpyrifos-methyl** | -4.9 | -5.9 | -5.1 | -5.4 | -4.8 | -5 | -5.3 | -4.6 | -5 | -4.4 | -5.9 | -5.7 | -4.5 | -5.3 | -5.3 | -4.8 | -5.7 | -5 | -5.5 | -4.9 | -6.2 | -5.3 | -4.7 | -4.9 | -5.3 | -4.5 | -4.9 | -5 |  |
| **Chlorthiophos** | -4.7 | -5.9 | -5 | -5 | -4.9 | -4.8 | -5.2 | -4.4 | -4.5 | -4.6 | -5.8 | -5.1 | -4.4 | -4.8 | -4.8 | -5.2 | -5.6 | -4.8 | -5.3 | -4.6 | -5.9 | -5.3 | -4.4 | -4.9 | -5.6 | -4.9 | -4.7 | -4.9 |  |
| **Coumaphos** | -6.4 | -6.5 | -6.4 | -6.9 | -5.4 | -5.6 | -5.6 | -5.4 | -6.1 | -5.4 | -6.5 | -6.7 | -4.3 | -6 | -6.1 | -5.5 | -6.1 | -6.2 | -6.2 | -5.3 | -5.8 | -6 | -5.5 | -6 | -6.5 | -5.6 | -6.8 | -5.5 |  |
| **Crotoxyphos** | -7 | -6.9 | -6 | -5.6 | -5.2 | -5.9 | -5.7 | -5.5 | -5.7 | -6 | -6.8 | -6.5 | -5.2 | -5.9 | -5.8 | -5.2 | -6.5 | -6 | -5.8 | -5.2 | -6.7 | -6 | -4.7 | -6 | -6 | -5.8 | -6.2 | -5.9 |  |
| **Crufomate** | -5.8 | -6 | -6 | -5.1 | -5.2 | -5.4 | -6.4 | -5.4 | -5.3 | -5.4 | -6.4 | -6.8 | -5.7 | -5.8 | -5.6 | -5.5 | -6.3 | -5.7 | -6 | -5.9 | -5.3 | -5.9 | -5.4 | -5.8 | -6.1 | -5.3 | -5.9 | -7 |  |
| **Cyanofenphos** | -6 | -6.6 | -5.6 | -5 | -5.3 | -5.6 | -7 | -6.2 | -5.7 | -5.2 | -6.6 | -7 | -5.1 | -6.2 | -5.4 | -5.5 | -5.6 | -5.5 | -5.6 | -5.6 | -6.7 | -5.3 | -4.9 | -5.7 | -7.4 | -5.6 | -6.3 | -5.5 |  |
| **Cyanophos** | -5.9 | -5.8 | -5.2 | -4.8 | -4.9 | -6.4 | -4.6 | -4.8 | -4.8 | -4.9 | -5.6 | -5.8 | -4.8 | -5 | -5.5 | -5.2 | -5.2 | -5 | -5.1 | -4.6 | -5.7 | -4.8 | -5.4 | -5 | -5.1 | -4.7 | -5.3 | -5.5 |  |
| **Cypermethrin** | -7.4 | -8.8 | -7.2 | -6.8 | -6.7 | -7.3 | -7.2 | -6.3 | -7.1 | -6.4 | -8.8 | -8.4 | -4.7 | -7.2 | -7.2 | -7.3 | -8.4 | -6.9 | -8.1 | -6.9 | -6.3 | -7 | -6.1 | -6 | -7.6 | -7.5 | -8 | -8.9 |  |
| **Demephion-O** | -3.3 | -4.1 | -3.8 | -3.2 | -3.3 | -3.4 | -3.3 | -3.5 | -3.4 | -3.3 | -4.1 | -3.6 | -5.8 | -3.7 | -3.6 | -3.7 | -3.7 | -3.6 | -3.4 | -3.7 | -3.6 | -3.5 | -3.1 | -3.4 | -3.4 | -3.7 | -3.3 | -3.8 |  |
| **Demephion-S** | -3.9 | -4.1 | -4 | -4.4 | -3.2 | -4 | -3.8 | -3.6 | -3.6 | -3.5 | -4 | -3.6 | -3.5 | -3.6 | -4 | -3.6 | -4 | -4.1 | -3.8 | -3.8 | -3.9 | -4 | -3.7 | -3.7 | -3.6 | -3.7 | -3.5 | -3.6 |  |
| **Demeton-O** | -3.8 | -4.6 | -4.1 | -3.6 | -3.9 | -3.8 | -4.1 | -3.8 | -3.9 | -3.8 | -4.6 | -4 | -3.7 | -3.6 | -4.1 | -4.6 | -4.3 | -3.8 | -3.7 | -3.7 | -4.4 | -4 | -3.5 | -3.9 | -4.1 | -3.8 | -4 | -3.8 |  |
| **Demeton-S** | -3.9 | -4.6 | -4.3 | -3.9 | -3.9 | -4.5 | -4.3 | -3.8 | -3.7 | -3.7 | -4.6 | -4.3 | -3.6 | -4.3 | -4.3 | -4.1 | -4.5 | -4.3 | -4.1 | -4.1 | -3.7 | -4.6 | -3.5 | -4.2 | -4.4 | -4 | -3.7 | -3.8 |  |
| **Demeton-S-methyl** | -4 | -4.4 | -4 | -4.7 | -3.7 | -4.1 | -3.8 | -3.7 | -3.8 | -3.5 | -4.3 | -3.7 | -3.7 | -3.6 | -3.9 | -3.6 | -3.9 | -4.1 | -3.9 | -3.6 | -4.1 | -4.1 | -3.8 | -3.9 | -3.9 | -3.5 | -3.4 | -4 |  |
| **Demeton-S-methylsulphon** | -4.4 | -4.9 | -4.8 | -5.1 | -4.1 | -4.6 | -4.2 | -3.9 | -4.5 | -3.9 | -4.8 | -4.3 | -4.2 | -4 | -4.7 | -4.1 | -4.8 | -5.1 | -4.6 | -4.1 | -4.8 | -4.5 | -3.9 | -4.4 | -4.5 | -4 | -3.9 | -4.7 |  |
| **Dialifos** | -6 | -6.6 | -6.4 | -6.2 | -5.6 | -6.3 | -7 | -5.4 | -5.3 | -5.1 | -6.6 | -6.6 | -4 | -5.9 | -6.3 | -5.7 | -6.4 | -5.8 | -5.5 | -6.2 | -5 | -6.3 | -4.9 | -5.4 | -6.9 | -5.8 | -6.5 | -5.1 |  |
| **Diazinon** | -5 | -6.3 | -6.3 | -5.6 | -5.6 | -5.4 | -5.6 | -5.1 | -5.1 | -5.2 | -6 | -6.1 | -5.2 | -5.5 | -6 | -5.2 | -6.3 | -5 | -5.4 | -5.6 | -6.3 | -6 | -5 | -5.8 | -5.8 | -6.1 | -5.5 | -6.1 |  |
| **Dichlofenthion** | -5.1 | -5.9 | -5.4 | -5.1 | -4.8 | -5.5 | -5.8 | -4.6 | -5.1 | -4.8 | -5.8 | -5.6 | -4.9 | -5.2 | -5.8 | -4.9 | -5.8 | -4.7 | -5.3 | -4.9 | -5.1 | -5.8 | -4.6 | -5.6 | -5.4 | -5 | -5.8 | -5.6 |  |
| **Dichlorvos** | -4.6 | -4.4 | -4.5 | -5.1 | -3.9 | -4.8 | -4.5 | -4.1 | -4.3 | -4 | -4.6 | -4.4 | -4.7 | -4.3 | -4.5 | -4 | -4.4 | -4.5 | -4.2 | -4.6 | -4.5 | -4.7 | -3.8 | -4.2 | -4.1 | -4 | -4.2 | -4.2 |  |
| **Dicrotophos** | -4.6 | -5.3 | -5.3 | -5.8 | -4.2 | -5 | -4.8 | -4.5 | -4.6 | -4.4 | -5.6 | -4.9 | -4.3 | -4.7 | -4.8 | -4.7 | -5 | -5.3 | -5.2 | -5 | -5.1 | -5.1 | -4.3 | -5.1 | -4.9 | -4.4 | -4.4 | -4.6 |  |
| **Dimefox** | -4 | -4.1 | -3.9 | -4.7 | -3.6 | -4.1 | -4.2 | -3.5 | -3.9 | -3.9 | -4.1 | -3.9 | -4.8 | -4.1 | -3.8 | -4.4 | -3.9 | -4 | -3.9 | -4.2 | -4.1 | -4.2 | -3.6 | -3.7 | -3.9 | -3.5 | -3.8 | -3.8 |  |
| **Dimethoate** | -3.9 | -4.5 | -4.5 | -4.2 | -4.2 | -4.6 | -3.9 | -3.7 | -3.8 | -3.7 | -4.5 | -4.1 | -3.5 | -3.9 | -4.1 | -5.2 | -4.4 | -4.2 | -4.4 | -4.7 | -4.3 | -4 | -3.7 | -4.5 | -4.2 | -3.7 | -3.7 | -4.6 |  |
| **Dioxabenzofos** | -6.7 | -5.7 | -5.2 | -5.6 | -4.7 | -6.1 | -6.1 | -4.5 | -5 | -4.8 | -6 | -6.3 | -3.8 | -5.2 | -6.1 | -5 | -5.7 | -4.9 | -5.3 | -5.6 | -6.2 | -6.1 | -5 | -4.8 | -5.6 | -5.5 | -5.1 | -5.4 |  |
| **Dioxathion** | -4.6 | -5.5 | -5.4 | -4.5 | -4.8 | -4.5 | -4.3 | -4.8 | -4.6 | -4.5 | -5.5 | -5.2 | -4.5 | -5 | -4.7 | -4.6 | -5.5 | -4.8 | -5.1 | -4.1 | -4.7 | -4.8 | -4.4 | -4.7 | -5.8 | -4.6 | -4.7 | -4.8 |  |
| **Disulfoton** | -3.7 | -4.7 | -4.2 | -3.7 | -3.7 | -3.7 | -3.9 | -3.7 | -3.7 | -3.5 | -4.6 | -3.8 | -4.2 | -3.9 | -4.2 | -3.8 | -4.2 | -3.9 | -3.9 | -4.1 | -4.4 | -4 | -3.6 | -3.7 | -4.1 | -3.6 | -4 | -4.1 |  |
| **Edifenphos** | -5.5 | -6.7 | -6 | -6.7 | -5.1 | -5.4 | -6.6 | -5 | -5.4 | -5.4 | -6.5 | -6.3 | -3.6 | -5.5 | -5.2 | -5.5 | -6.5 | -5.7 | -5.9 | -5.9 | -6.8 | -6.3 | -4.8 | -5.7 | -6.5 | -6.2 | -5.6 | -5.4 |  |
| **EPBP** | -6.3 | -6.8 | -6.4 | -6.1 | -5.9 | -6.3 | -6.4 | -5.8 | -6 | -5.5 | -6.9 | -6.9 | -5.1 | -6.5 | -6 | -5.7 | -7 | -5.7 | -5.9 | -5.6 | -7.3 | -6.7 | -5 | -5.9 | -7 | -5.9 | -6.5 | -5.4 |  |
| **EPN** | -6.1 | -7 | -6.2 | -6.6 | -6.3 | -6.5 | -6.4 | -6.2 | -5.7 | -6.1 | -7.2 | -6.9 | -5.6 | -6.6 | -5.9 | -5.9 | -6 | -6 | -6 | -5.8 | -7.1 | -6.3 | -5 | -6.3 | -7.2 | -6 | -6.9 | -7.4 |  |
| **Ethion** | -4.1 | -4.8 | -4.7 | -3.9 | -4.2 | -4.4 | -4.2 | -4 | -4.3 | -3.9 | -4.8 | -4.5 | -5.2 | -4.3 | -4 | -4.3 | -4.5 | -4.3 | -4.3 | -3.7 | -5.1 | -4.2 | -3.8 | -4.2 | -4.9 | -4 | -4.4 | -4.2 |  |
| **Ethoprophos** | -4 | -4.7 | -4.3 | -5.3 | -3.9 | -4.8 | -4.2 | -4 | -3.8 | -3.6 | -4.8 | -4.5 | -3.7 | -4 | -4.1 | -3.9 | -4.6 | -4.2 | -4 | -4 | -3.8 | -4.8 | -3.8 | -4.7 | -4.4 | -4.2 | -4.2 | -4 |  |
| **Famphur** | -5.1 | -6 | -5.6 | -6.4 | -4.8 | -5.2 | -5.1 | -5.2 | -4.9 | -5.1 | -5.6 | -6.5 | -3.6 | -5.7 | -4.9 | -5.2 | -5.7 | -5.6 | -5.7 | -5.1 | -6.1 | -5.2 | -4.8 | -5.3 | -5.9 | -5 | -4.8 | -5.3 |  |
| **Fenamiphos** | -5.3 | -6.7 | -5.5 | -6.4 | -5.1 | -5.9 | -5.6 | -5.3 | -5.5 | -5 | -6.2 | -6.5 | -4.7 | -5.8 | -5.6 | -5.2 | -6.1 | -5.5 | -5.7 | -5.7 | -6.4 | -6 | -5 | -5.6 | -6.1 | -5.8 | -6.1 | -6 |  |
| **Fenchlorphos** | -4.6 | -6.4 | -5.1 | -5 | -4.5 | -5 | -5.4 | -4.7 | -4.7 | -4.4 | -5.9 | -5.9 | -5.2 | -5.1 | -5.2 | -4.8 | -5.5 | -4.9 | -5.2 | -4.6 | -6 | -5.9 | -4.5 | -5.2 | -5.2 | -4.8 | -4.9 | -5 |  |
| **Fenitrothion** | -5.8 | -5.8 | -5.2 | -6.4 | -5.3 | -5.1 | -5.7 | -5.1 | -5.3 | -5.2 | -6.4 | -6.4 | -4.5 | -5.8 | -6.3 | -5.2 | -5.5 | -5.2 | -6 | -5.1 | -5.9 | -5.4 | -5.6 | -5.6 | -5.7 | -5 | -5.2 | -6.5 |  |
| **Fensulfothion** | -5 | -6.2 | -5.7 | -6.6 | -4.8 | -5.9 | -5.4 | -5 | -5 | -4.8 | -5.8 | -6.1 | -4.9 | -5.8 | -5.2 | -4.8 | -5.4 | -5 | -5.5 | -5.5 | -6 | -5.3 | -4.9 | -5.3 | -5.5 | -5.4 | -5.2 | -6 |  |
| **Fenthion** | -5 | -5.8 | -4.9 | -5.8 | -4.4 | -5.1 | -5.2 | -4.8 | -4.5 | -4.7 | -5.7 | -5.9 | -4.7 | -5.2 | -5.9 | -4.7 | -5.1 | -4.9 | -5.1 | -5 | -5.5 | -4.9 | -4.6 | -5 | -5.4 | -4.8 | -4.9 | -5.5 |  |
| **Fonofos** | -4.3 | -5.2 | -4.5 | -4.4 | -4.3 | -4.6 | -4.9 | -4.1 | -4.6 | -4.2 | -4.9 | -5.5 | -4.7 | -4.5 | -4.1 | -4.8 | -5.2 | -4.7 | -4.8 | -4.6 | -5.4 | -4.9 | -4.1 | -4.6 | -4.7 | -5.1 | -4.4 | -4.7 |  |
| **Formothion** | -4.2 | -4.8 | -5 | -4.6 | -3.7 | -4.3 | -4.6 | -4 | -4 | -4.3 | -4.9 | -4.3 | -4.2 | -4 | -4.5 | -4.3 | -4.7 | -4.6 | -5 | -4.2 | -4.6 | -4.6 | -3.9 | -4.2 | -4.5 | -3.7 | -3.9 | -4.3 |  |
| **Fosmethilan** | -6 | -5.7 | -5.7 | -4.9 | -5 | -5.6 | -4.9 | -4.9 | -5.1 | -5.4 | -6.6 | -5.5 | -3.9 | -5.4 | -4.9 | -5.4 | -6.4 | -5.5 | -5.4 | -4.9 | -6.2 | -5.7 | -4.9 | -5 | -6.5 | -5 | -5.3 | -5.3 |  |
| **glyphosate** | -4.9 | -5.5 | -5 | -4.9 | -4.3 | -5.1 | -4.7 | -4.5 | -4.5 | -4.7 | -4.7 | -4.9 | -4.5 | -4.2 | -4.7 | -5.6 | -4.7 | -4.8 | -5.1 | -4.9 | -4.1 | -4.5 | -4.6 | -5 | -4.9 | -4.4 | -4.3 | -5 |  |
| **Heptenophos** | -4.9 | -5.5 | -5 | -5 | -5.2 | -5.6 | -5.8 | -4.6 | -4.8 | -4.5 | -6 | -6 | -4.7 | -5.5 | -5.6 | -5.9 | -6.2 | -5.8 | -5.2 | -5.3 | -6 | -5.5 | -4.6 | -5.2 | -5.4 | -4.6 | -5 | -5.2 |  |
| **Pirimiphos-methyl** | -4.9 | -5.6 | -5.5 | -5.9 | -5.5 | -5.3 | -5.2 | -5 | -5 | -4.6 | -6.5 | -5.7 | -4.2 | -5.1 | -5.6 | -4.9 | -5.8 | -5.5 | -5.8 | -5.5 | -6.5 | -5.8 | -4.8 | -6 | -5.6 | -4.9 | -5.4 | -5.9 |  |
| **Isazofos** | -5 | -5.7 | -5 | -4.5 | -5 | -4.7 | -5.6 | -4.7 | -5.2 | -4.4 | -6 | -5.9 | -5.1 | -4.9 | -5.9 | -4.7 | -5.7 | -5.3 | -5.1 | -4.7 | -5.8 | -5.3 | -4.9 | -5 | -5.8 | -4.9 | -4.8 | -5.5 |  |
| **Isofenphos** | -5.4 | -6.2 | -5.9 | -6.1 | -5.7 | -5.9 | -6.3 | -5.3 | -5.2 | -5.5 | -6.2 | -5.6 | -3.8 | -5.4 | -5.8 | -5.8 | -6.1 | -5.2 | -5.5 | -6 | -6.6 | -6.6 | -5 | -5.4 | -6 | -5.5 | -5.6 | -5.3 |  |
| **Isothioate** | -3.7 | -4.3 | -4.1 | -3.5 | -3.5 | -4 | -3.7 | -3.7 | -3.6 | -3.5 | -4.5 | -3.9 | -5.3 | -3.8 | -4 | -4 | -4.2 | -3.9 | -4 | -3.3 | -4.4 | -4.1 | -3.6 | -3.8 | -3.9 | -3.6 | -3.8 | -4.1 |  |
| **Isoxathion** | -6.7 | -6.8 | -7 | -7.2 | -5.7 | -6.5 | -6.6 | -5.8 | -5.9 | -5.8 | -6.4 | -6.6 | -4.2 | -5.9 | -6.1 | -5.8 | -6.4 | -6.2 | -6.1 | -6.3 | -7.4 | -5.8 | -5.4 | -5.9 | -6.2 | -5.7 | -6.2 | -6.6 |  |
| **Jodfenphos** | -4.6 | -4.9 | -5.2 | -4.6 | -4 | -4.7 | -4.5 | -4.5 | -4.5 | -4.3 | -5.6 | -5.9 | -5.2 | -5 | -4.7 | -4.7 | -5.3 | -4.5 | -4.7 | -4.6 | -4.5 | -4.4 | -4.6 | -4.5 | -5.1 | -4.3 | -4.9 | -4.8 |  |
| **Leptophos** | -6 | -7.2 | -6.3 | -5.8 | -5.5 | -6.5 | -7 | -5.5 | -6.3 | -5.5 | -7.1 | -7 | -4.2 | -6.1 | -6 | -5.8 | -7 | -5.8 | -6.6 | -5.7 | -7.6 | -6.7 | -5.3 | -6 | -6.8 | -6.2 | -6.7 | -6.8 |  |
| **Malathion** | -4.5 | -5.1 | -5 | -5.5 | -4.1 | -5.1 | -4.8 | -4.1 | -4.3 | -4.4 | -5.6 | -4.8 | -5.3 | -4.5 | -4.9 | -4.7 | -5.2 | -5.1 | -4.9 | -4.8 | -5.4 | -4.8 | -4.8 | -4.9 | -4.8 | -4.5 | -4.4 | -4.4 |  |
| **Menazon** | -4.8 | -6.6 | -5.8 | -6.2 | -5.2 | -5.8 | -5.1 | -5.5 | -5 | -4.8 | -6 | -5.6 | -4.5 | -5 | -5.3 | -5.9 | -6.3 | -5.7 | -5.9 | -6.3 | -5.7 | -5.8 | -5 | -5.8 | -5.6 | -5.1 | -5.6 | -5.6 |  |
| **Mephosfolan** | -4.7 | -5.4 | -4.6 | -5.7 | -4.2 | -5.6 | -4.8 | -4.5 | -5.2 | -4.2 | -5.3 | -5 | -4.6 | -4.5 | -5.1 | -4.7 | -5.4 | -4.8 | -4.9 | -4.7 | -5.3 | -4.8 | -4.4 | -5.1 | -5.1 | -4.3 | -4.7 | -4.7 |  |
| **Methacrifos** | -4.5 | -4.8 | -5.1 | -4.5 | -4.3 | -4.8 | -4.7 | -4.1 | -4.3 | -4.2 | -5 | -4.6 | -3.6 | -4.9 | -4.9 | -4.4 | -4.9 | -4.8 | -4.9 | -4.3 | -4.8 | -4.9 | -4.3 | -4.6 | -4.8 | -4.3 | -4.1 | -4.6 |  |
| **Methamidophos** | -4.1 | -3.7 | -4.1 | -3.9 | -3.4 | -3.8 | -3.8 | -3.6 | -3.3 | -3.9 | -3.7 | -3.7 | -4.4 | -3.6 | -3.6 | -3.5 | -3.6 | -3.9 | -4 | -3.7 | -3.4 | -3.8 | -3.2 | -3.7 | -3.5 | -3.7 | -3.8 | -3.7 |  |
| **Methidathion** | -4.6 | -4.7 | -5.3 | -5.2 | -4.6 | -4.8 | -4.7 | -4.5 | -4.7 | -4.1 | -5.2 | -4.9 | -4.6 | -4.7 | -5 | -4.4 | -4.5 | -4.7 | -4.4 | -5.5 | -4.2 | -4.6 | -3.9 | -4.2 | -5 | -4.5 | -4.1 | -4.8 |  |
| **Mevinphos** | -4.7 | -5 | -5 | -5.6 | -4.2 | -5.3 | -4.6 | -4.2 | -4.6 | -4.3 | -5 | -4.7 | -4.5 | -4.7 | -4.8 | -4.7 | -5.2 | -5 | -4.9 | -4.2 | -4.8 | -4.7 | -4.6 | -4.8 | -4.5 | -4.2 | -4.2 | -4.8 |  |
| **Monocrotophos** | -4.8 | -5.1 | -5 | -5.5 | -4.2 | -4.9 | -4.8 | -4.6 | -4.7 | -4.3 | -5.3 | -5.1 | -4.2 | -4.8 | -5.1 | -5 | -5.1 | -4.8 | -4.8 | -5.1 | -5.1 | -5.1 | -4.4 | -5 | -4.6 | -4.5 | -4.8 | -4.7 |  |
| **Naled** | -4.3 | -4.4 | -4.8 | -4.8 | -4.1 | -4.2 | -5 | -4.3 | -4.2 | -4.3 | -5 | -4.6 | -4.1 | -4.7 | -4.2 | -4.4 | -5.1 | -4.5 | -4 | -4.6 | -4.8 | -4.1 | -4 | -4.6 | -4.8 | -4.2 | -4.2 | -4.2 |  |
| **Omethoate** | -4.3 | -4.5 | -4.7 | -4.6 | -4 | -4.5 | -4.5 | -3.8 | -4 | -3.9 | -4.8 | -4.3 | -4.1 | -4 | -4.1 | -4.2 | -4.7 | -4.5 | -4.5 | -4.5 | -4.3 | -4.4 | -4.5 | -4.1 | -3.9 | -4 | -3.9 | -4.3 |  |
| **Parathion** | -5.7 | -5.9 | -5.6 | -5.8 | -5.1 | -6.2 | -5.5 | -5.2 | -5.2 | -4.9 | -6 | -6.1 | -4.8 | -5.6 | -5.5 | -5.3 | -5.8 | -5.1 | -5.7 | -5.7 | -5.9 | -6.2 | -4.8 | -5.4 | -5.8 | -5.2 | -5.9 | -5 |  |
| **Parathion-methyl** | -5.7 | -5.9 | -5.6 | -5.8 | -4.9 | -5.9 | -5.4 | -4.9 | -5.4 | -5.1 | -6.2 | -5.9 | -5.2 | -5.3 | -5.5 | -5.2 | -5.7 | -5 | -5.7 | -6 | -5.8 | -6.1 | -5.2 | -5.2 | -5.5 | -5.1 | -4.9 | -6 |  |
| **Phenthoate** | -5.8 | -6.1 | -5.3 | -5.3 | -5 | -5.9 | -5.6 | -4.6 | -5.1 | -5.2 | -5.9 | -6 | -5.1 | -5.3 | -5.8 | -4.9 | -6.1 | -5.7 | -5.3 | -5.5 | -5.4 | -5.8 | -4.7 | -5.3 | -5.6 | -5.1 | -5.3 | -6 |  |
| **Phorate** | -3.7 | -4.6 | -3.6 | -3.5 | -3.5 | -4.4 | -3.9 | -3.7 | -3.6 | -3.4 | -4.5 | -3.7 | -3.3 | -3.7 | -3.7 | -3.9 | -4.2 | -3.7 | -3.9 | -3.7 | -4.5 | -3.8 | -3.4 | -3.8 | -4.1 | -3.5 | -3.4 | -3.5 |  |
| **Phosalone** | -5.2 | -5.7 | -5.6 | -6.1 | -5.4 | -5.7 | -5.4 | -4.9 | -5.2 | -4.5 | -6.3 | -5.9 | -4.5 | -5.2 | -5.6 | -7.2 | -5 | -5.6 | -5.3 | -4.8 | -5.2 | -4.8 | -4.8 | -5.1 | -6 | -5.3 | -6.1 | -5 |  |
| **Phosmet** | -6.8 | -6.7 | -6.8 | -7.2 | -5.3 | -6.4 | -6.4 | -5.5 | -5.4 | -5.2 | -6.7 | -6.6 | -5.3 | -5.6 | -6.5 | -5.6 | -6.3 | -5.9 | -6.1 | -6.3 | -6.9 | -6 | -5 | -5.6 | -6.2 | -5.4 | -5.9 | -5.5 |  |
| **Phosphamidon** | -4.6 | -5.2 | -5.5 | -5.7 | -4.5 | -4.6 | -4.8 | -4.7 | -4.4 | -4.7 | -5.8 | -4.8 | -4.5 | -4.7 | -4.9 | -4.5 | -5.4 | -4.7 | -5.3 | -4.3 | -5.5 | -5.2 | -4.4 | -5 | -5.3 | -4.2 | -4.9 | -5.6 |  |
| **Phospholan** | -4.3 | -5.1 | -4.9 | -5.7 | -4.5 | -5 | -4.7 | -4.3 | -5.1 | -4 | -5.1 | -4.9 | -4.5 | -4.4 | -4.9 | -4.9 | -5 | -4.6 | -4.8 | -4.5 | -5.3 | -5 | -4.1 | -4.8 | -4.7 | -4.5 | -4.7 | -4.6 |  |
| **Phoxim** | -6.1 | -6.6 | -6.5 | -6.1 | -5.3 | -6.2 | -6.1 | -5.4 | -6.1 | -5.6 | -6.5 | -6.3 | -5.2 | -5.8 | -6.4 | -5.7 | -6.1 | -5.6 | -6 | -6.3 | -5.4 | -6.3 | -5.5 | -5.6 | -6.2 | -5.6 | -6 | -6.5 |  |
| **Pirimiphos-ethyl** | -5 | -6 | -5.6 | -5.8 | -5.7 | -5.6 | -5.2 | -4.7 | -5.3 | -4.7 | -5.9 | -6 | -4.9 | -5 | -5.8 | -5 | -5.8 | -5.4 | -5.2 | -5.3 | -6.4 | -5.7 | -4.7 | -5.8 | -5.8 | -5.1 | -5.1 | -5.1 |  |
| **Profenofos** | -5 | -5.9 | -5.4 | -5.1 | -5 | -5.2 | -5.4 | -4.4 | -5.1 | -4.6 | -6.1 | -6.3 | -5.1 | -5.2 | -5.8 | -4.5 | -5.5 | -4.8 | -5.2 | -4.9 | -4.7 | -5.5 | -4.5 | -5.5 | -5.4 | -5.4 | -5.2 | -5 |  |
| **Propaphos** | -5.2 | -6.2 | -5.6 | -6.9 | -5.2 | -5.9 | -5.5 | -5.1 | -5 | -4.6 | -5.9 | -5.8 | -4.4 | -5.2 | -5.5 | -4.6 | -5.5 | -5.2 | -5.3 | -5.7 | -5.7 | -5.8 | -4.9 | -5.4 | -5.4 | -5.2 | -5.9 | -5.3 |  |
| **Propetamphos** | -4.4 | -5.7 | -5.6 | -4.6 | -4.3 | -5.1 | -5.3 | -4.5 | -4.5 | -4.8 | -5.6 | -4.9 | -4.7 | -5 | -5.1 | -5 | -5.2 | -4.9 | -5.1 | -4.4 | -4.4 | -4.9 | -4.2 | -4.4 | -5.1 | -4.9 | -4.4 | -5 |  |
| **Prothiofos** | -4.8 | -5.4 | -5.2 | -5 | -4.6 | -4.7 | -5.3 | -4.8 | -4.8 | -4.5 | -5.8 | -5.2 | -4.7 | -5.2 | -5.5 | -4.5 | -5.5 | -4.6 | -5.1 | -5 | -5.5 | -5.9 | -4.3 | -5.3 | -5.7 | -5.3 | -5.2 | -5.7 |  |
| **Prothoate** | -4.4 | -5.3 | -5.2 | -4.5 | -4.5 | -4.2 | -5.1 | -4.3 | -4.3 | -4.2 | -5.3 | -4.7 | -4.5 | -4.6 | -4.6 | -4.7 | -5 | -4.5 | -4.8 | -4.2 | -5.1 | -4.6 | -3.9 | -4.6 | -5 | -4.2 | -4.4 | -4.9 |  |
| **Pyraclofos** | -6.1 | -6.7 | -6.7 | -7.1 | -5.5 | -6.6 | -6 | -5.7 | -6 | -5.1 | -6.9 | -6.5 | -4.5 | -5.9 | -6.1 | -6.1 | -6.8 | -5.6 | -6.3 | -6.2 | -7.4 | -6.4 | -5.4 | -6.1 | -6.3 | -5.4 | -6.3 | -6.2 |  |
| **Pyridaphenthion** | -5.2 | -7.1 | -5.6 | -7.2 | -5.2 | -6.2 | -7.2 | -5.7 | -5.5 | -5.1 | -6.9 | -6.8 | -5.2 | -6 | -6.9 | -5.7 | -6.8 | -6 | -6 | -5.6 | -7.3 | -6 | -5.2 | -6 | -6.5 | -5.6 | -6.8 | -5.6 |  |
| **Schradan** | -3.8 | -4.7 | -4.6 | -4.2 | -4.3 | -4.3 | -3.8 | -3.8 | -4.3 | -4.1 | -5.2 | -4.6 | -5.8 | -4.6 | -3.8 | -4.4 | -5.1 | -4.9 | -4.7 | -4.1 | -4 | -4.7 | -4.3 | -4.4 | -4.7 | -3.7 | -4 | -4.5 |  |
| **Sulfotep** | -4 | -4.8 | -4.8 | -4.2 | -4.2 | -4 | -4.8 | -4.3 | -4.2 | -4.5 | -5.2 | -4.2 | -4.2 | -4.4 | -4.4 | -4.1 | -5 | -4.4 | -4.6 | -4.4 | -4.9 | -4.7 | -4 | -4.3 | -4.5 | -4.1 | -4 | -4.4 |  |
| **Sulprofos** | -4.9 | -5.6 | -4.6 | -4.7 | -4.8 | -4.9 | -4.3 | -4.7 | -4.7 | -4 | -5.4 | -5.5 | -4 | -4.7 | -4.9 | -4.2 | -5 | -4.8 | -4.6 | -4.5 | -5.3 | -5.3 | -4 | -5.1 | -5.2 | -4.9 | -4.9 | -4.4 |  |
| **Temephos** | -5.9 | -6.6 | -6.1 | -5.1 | -5.3 | -6.2 | -5.7 | -5.6 | -6.1 | -5.6 | -6.3 | -6.8 | -4.4 | -5.9 | -5.2 | -5.8 | -5.7 | -5.8 | -6.1 | -5 | -5.2 | -5.2 | -5.1 | -5.5 | -6.1 | -6 | -6.3 | -5.5 |  |
| **TEPP** | -4.6 | -5 | -4.9 | -5.2 | -4.4 | -5.2 | -4.8 | -4.6 | -4.5 | -4.2 | -5.1 | -4.8 | -4.1 | -4.7 | -4.4 | -4.7 | -5.2 | -5.1 | -4.8 | -4.7 | -5.1 | -4.9 | -4.2 | -4.8 | -4.9 | -4.4 | -4.5 | -4.4 |  |
| **Terbufos** | -3.9 | -4.7 | -4.2 | -3.7 | -4 | -4.2 | -4.7 | -3.9 | -3.9 | -3.8 | -4.8 | -4.3 | -5.9 | -4.2 | -4 | -4.3 | -4.5 | -4 | -4.4 | -3.8 | -5.1 | -4 | -3.9 | -3.9 | -4.7 | -3.6 | -4.4 | -4 |  |
| **Tetrachlorvinphos** | -5.5 | -6.3 | -5.1 | -5.1 | -5 | -5.3 | -5.6 | -4.7 | -5 | -5.1 | -6.2 | -5.9 | -3.8 | -5.5 | -5.3 | -5.6 | -6.1 | -5.5 | -5.1 | -4.7 | -6.2 | -5.6 | -4.9 | -5.4 | -5.9 | -5.1 | -5.9 | -5.3 |  |
| **Thiometon** | -3.6 | -4.2 | -4 | -3.7 | -3.2 | -4 | -3.6 | -3.4 | -3.5 | -3.4 | -4.2 | -3.7 | -4.5 | -3.6 | -3.8 | -3.6 | -3.9 | -3.9 | -3.7 | -4.1 | -4 | -3.9 | -3.2 | -3.6 | -3.5 | -3.4 | -3.3 | -3.5 |  |
| **Thionazin** | -4.5 | -5.3 | -5.3 | -6 | -5.2 | -5 | -5.1 | -4.4 | -4.9 | -4.4 | -5.5 | -4.7 | -3.4 | -4.7 | -4.8 | -5.4 | -5.1 | -5.1 | -5.3 | -4.6 | -5 | -5.1 | -4 | -5 | -5 | -4.4 | -4.1 | -4.9 |  |
| **Triazophos** | -6.4 | -6.7 | -7 | -6.8 | -5.6 | -6.2 | -6.2 | -5.4 | -5.8 | -5.7 | -6.2 | -6.5 | -4.2 | -5.5 | -5.8 | -5.6 | -6.7 | -6.5 | -6.1 | -7 | -5.6 | -6.3 | -5.4 | -6.1 | -5.9 | -5.8 | -6.3 | -5.8 |  |
| **Trichlorfon** | -4.7 | -4.4 | -4.9 | -4.4 | -4.2 | -4.4 | -5 | -4.1 | -4.5 | -4 | -4.8 | -4.2 | -5.2 | -4.3 | -4.1 | -4.8 | -4.6 | -4.5 | -4.7 | -4.9 | -4.6 | -4.7 | -4.3 | -4.3 | -4.2 | -4.1 | -4.3 | -4.3 |  |
| **Vamidothion** | -4.3 | -4.9 | -5.1 | -4.9 | -4.2 | -4.6 | -5.1 | -4.5 | -4.3 | -4.3 | -4.9 | -4.7 | -3.8 | -4.4 | -4.4 | -4.3 | -4.6 | -4.5 | -4.7 | -4.1 | -5 | -4.5 | -3.8 | -4.6 | -4.8 | -4.1 | -4.2 | -4.6 |  |
| **Oxydemeton-methyl** | -4.9 | -4.3 | -4.3 | -3.9 | -4 | -4.6 | -4.2 | -3.7 | -4 | -3.9 | -4.6 | -4 | -4.5 | -4 | -4.4 | -4.9 | -4.5 | -4.3 | -4.3 | -3.9 | -4.3 | -4.4 | -3.8 | -4.1 | -4.3 | -3.8 | -3.5 | -4.1 |  |
| **Alachlor** | -5.1 | -5.5 | -5.3 | -4.9 | -4.9 | -5.2 | -5.7 | -4.5 | -5.2 | -4.8 | -5.6 | -5.5 | -4.7 | -4.2 | -5.3 | -5.3 | -5.9 | -5.2 | -5 | -5.3 | -5.6 | -6 | -4.7 | -5.5 | -5.5 | -5 | -5.7 | -5.2 |  |
| **DFP** | -5.4 | -5.9 | -5.5 | -5.8 | -5.5 | -5.0 | -4.8 | -3.5 | -5.5 | -5.4 | -4.6 | -5.6 | -4.2 | -5.5 | -4.2 | -5.3 | -5.3 | -4.8 | -4.3 | -5.4 | -5.5 | -4.4 | -5.5 | -4.6 | -3.5 | -4.6 | -4.4 | -4.6 | |
| **Cyclosarin** | -4.1 | -4.2 | -4.3 | -4.3 | -4.3 | -5.2 | -4.7 | -5 | -4.3 | -4.7 | -3.9 | -4.8 | -4.3 | -5.1 | -3.9 | -4.8 | -5.3 | -4.7 | -4 | -4.7 | -4.8 | -4 | -4.3 | -5.2 | -5 | -5.2 | -4 | -4.6 | |
| **Sarin** | -4.5 | -4.8 | -4.4 | -4.6 | -4.4 | -4.0 | -5.3 | -5.5 | -4.4 | -4.4 | -5.5 | -4.3 | -4.8 | -3.7 | -5.1 | -4.3 | -3.7 | -5.6 | -4 | -4.4 | -3.9 | -5.3 | -4.4 | -5.0 | -5.5 | -5.0 | -5.3 | -5.2 | |
| **Soman** | -3.7 | -4.3 | -4 | -4.1 | -4 | -4.9 | -3.6 | -5.6 | -4 | -5.1 | -3.6 | -4.9 | -5.1 | -5.2 | -3.6 | -4.9 | -5.2 | -3.6 | -5.1 | -5.1 | -5.4 | -3.5 | -4 | -5.2 | -5.6 | -5.2 | -3.5 | -5.0 | |
| **Tabun** | -4.5 | -5.5 | -4.2 | -5.2 | -4.2 | -4.6 | -5.1 | -4.6 | -4.2 | -4.5 | -4.5 | -5.3 | -5.2 | -5.6 | -4.5 | -5.3 | -5.6 | -5.1 | -4.2 | -4.5 | -3.6 | -4.9 | -4.2 | -4.0 | -4.6 | -4.0 | -4.9 | -5.2 | |


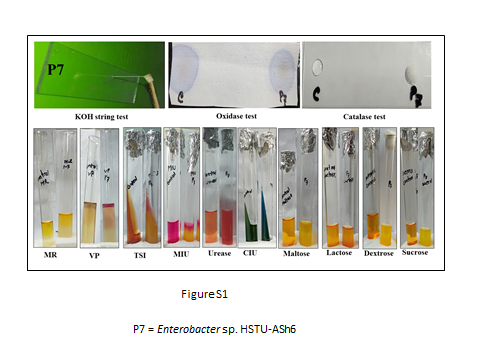


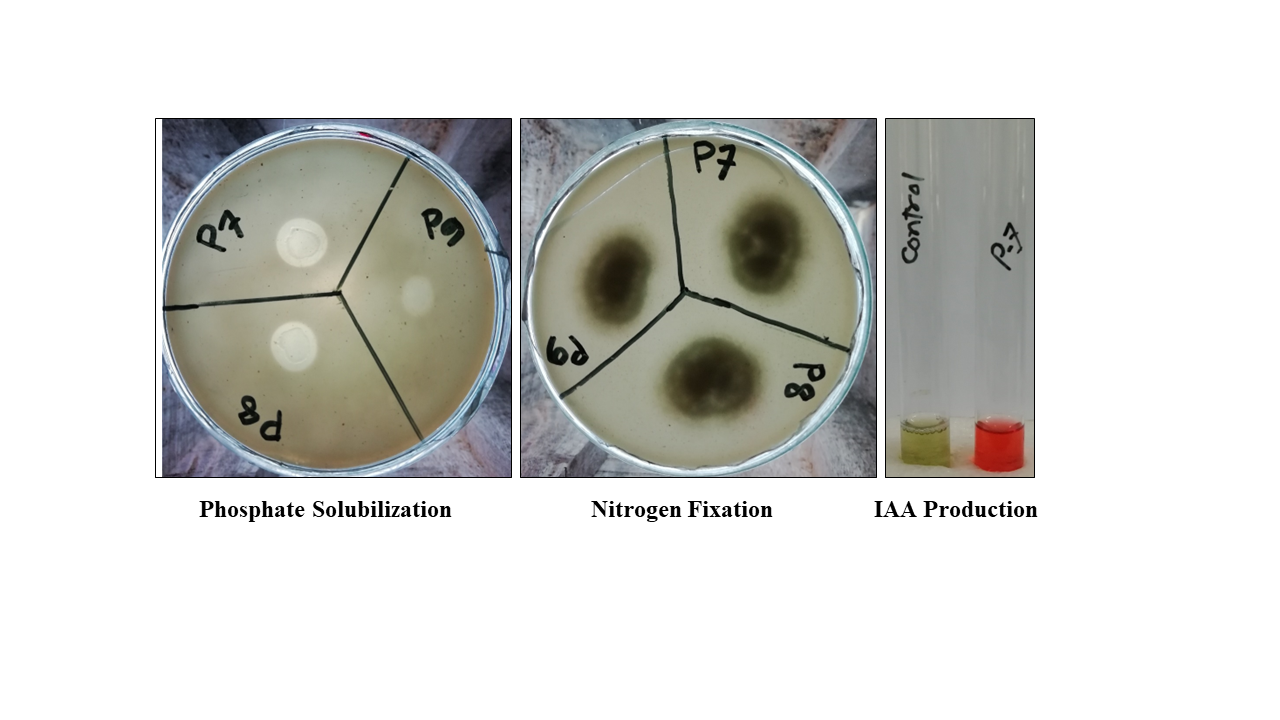

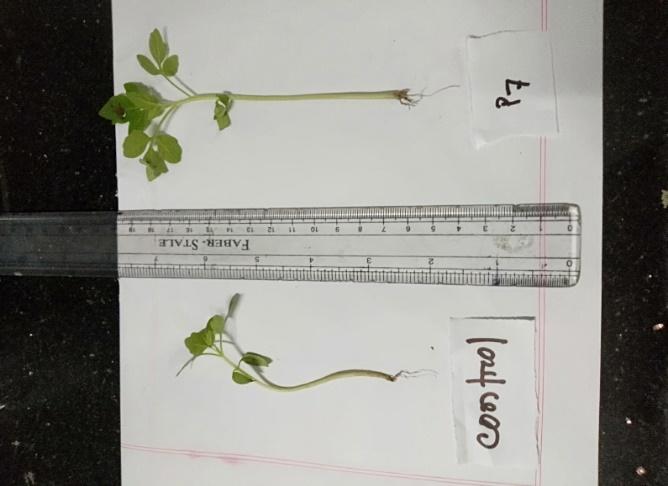

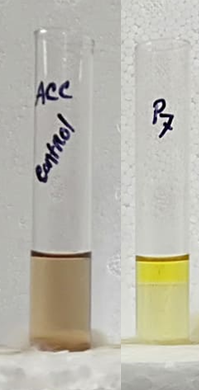


**ACC-deaminase**

P7 = *Enterobacter* sp. HSTU-ASh6

**Figure S2**


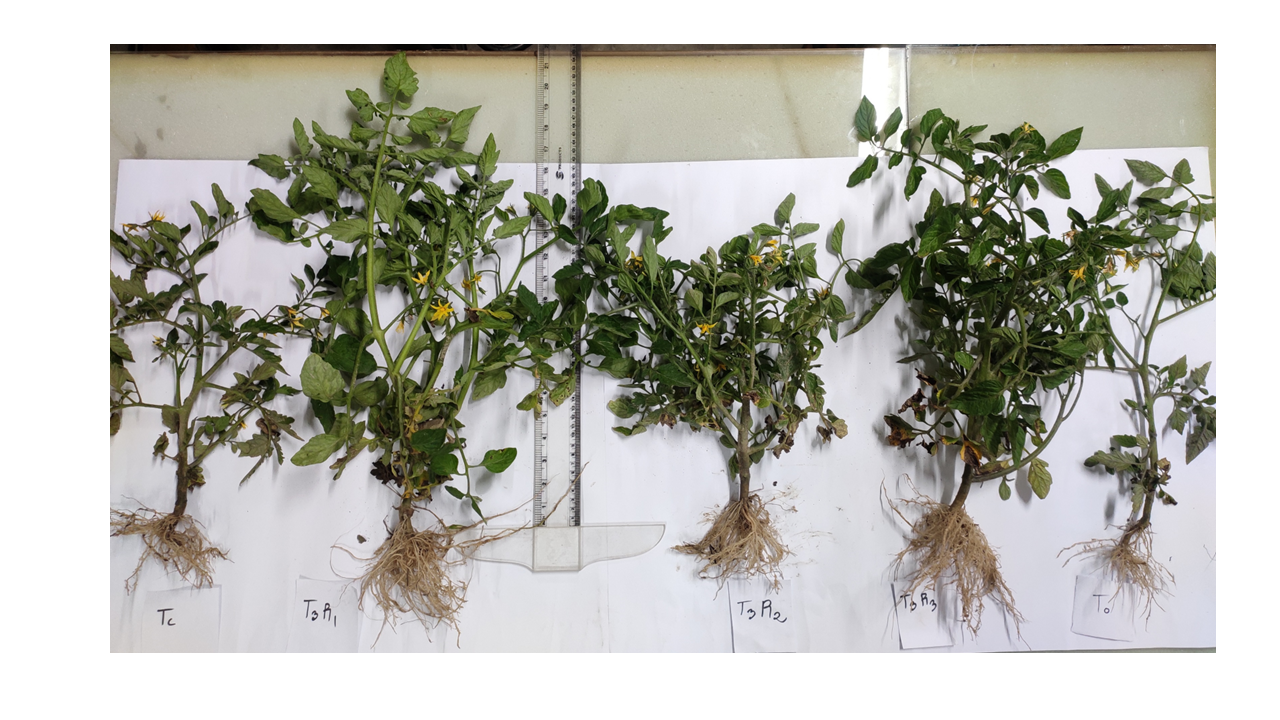

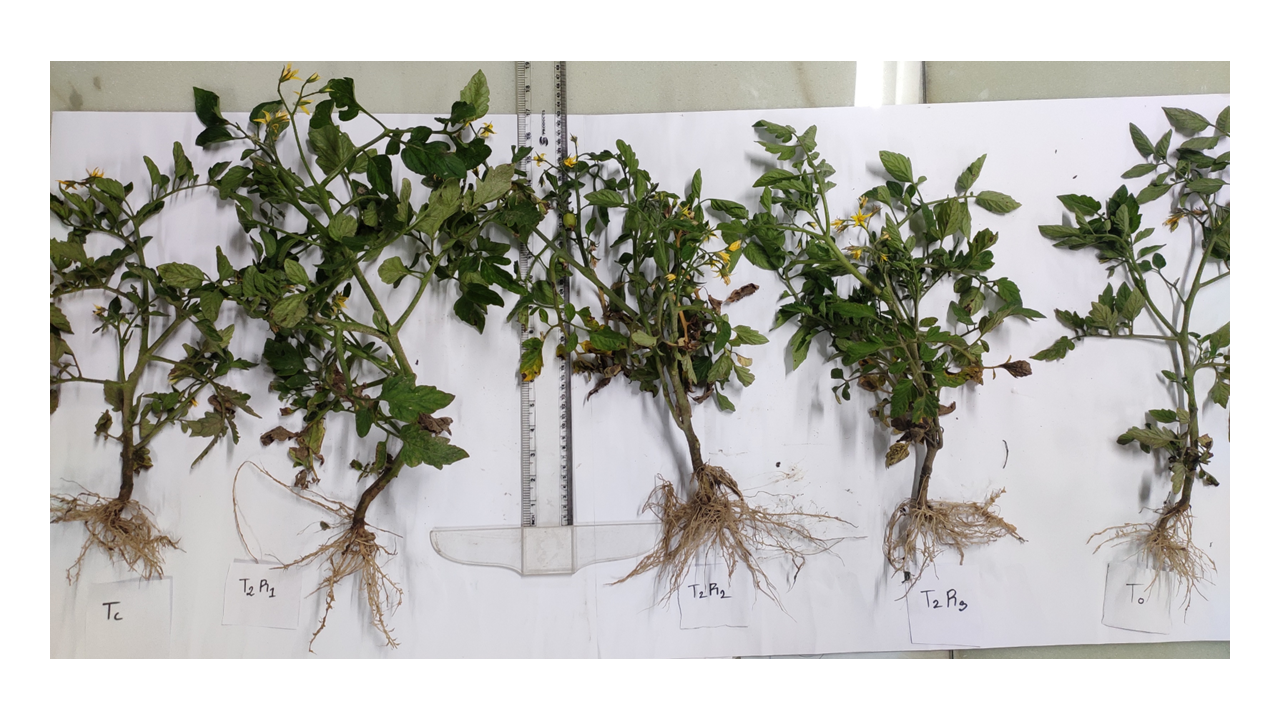

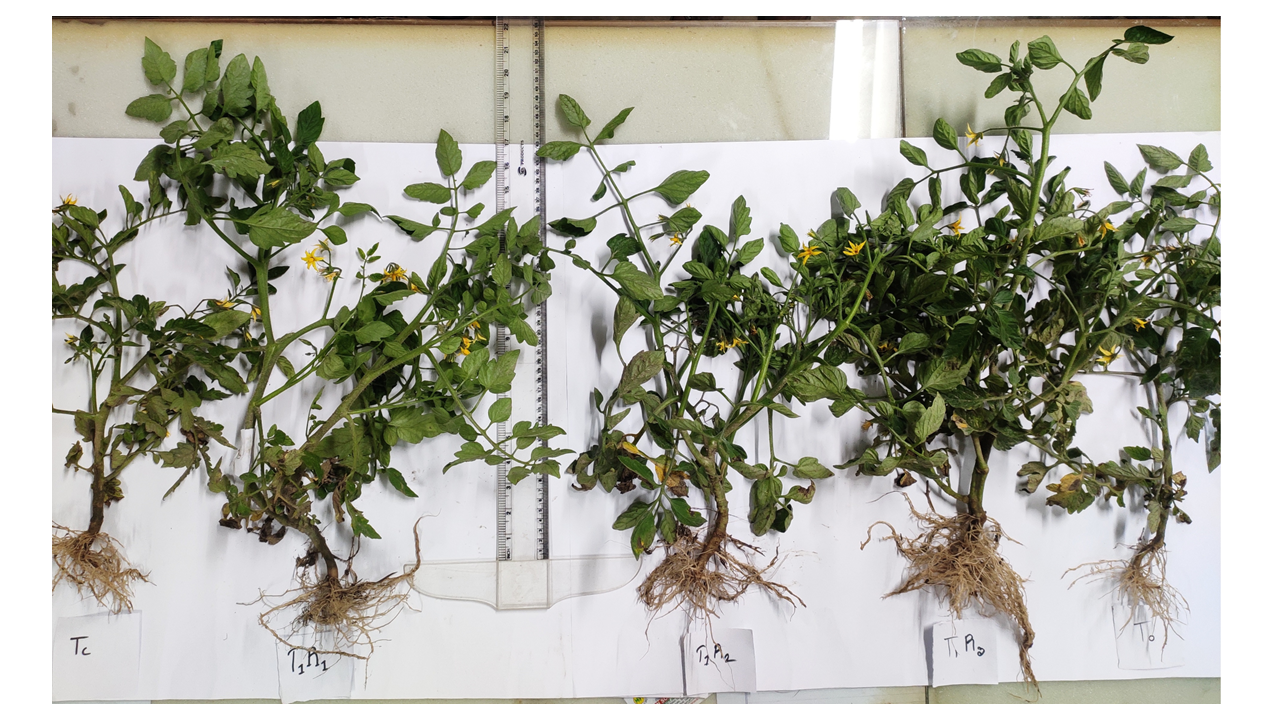


Figure S3A. Tomato plants after 30 Days of plantation.

**30% urea+ HSTU-ASh6 treatment**

**70% urea+ HSTU-ASh6 treatment**

**100% urea+ HSTU-ASh6 treatment**


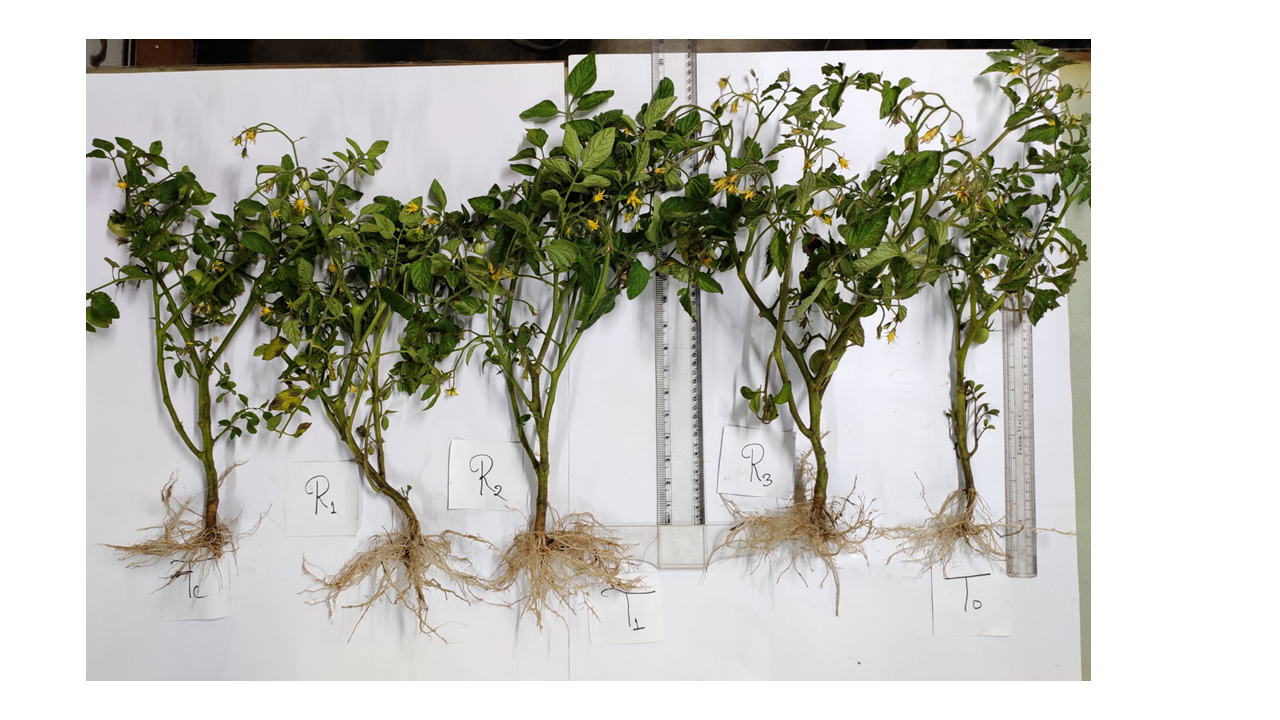

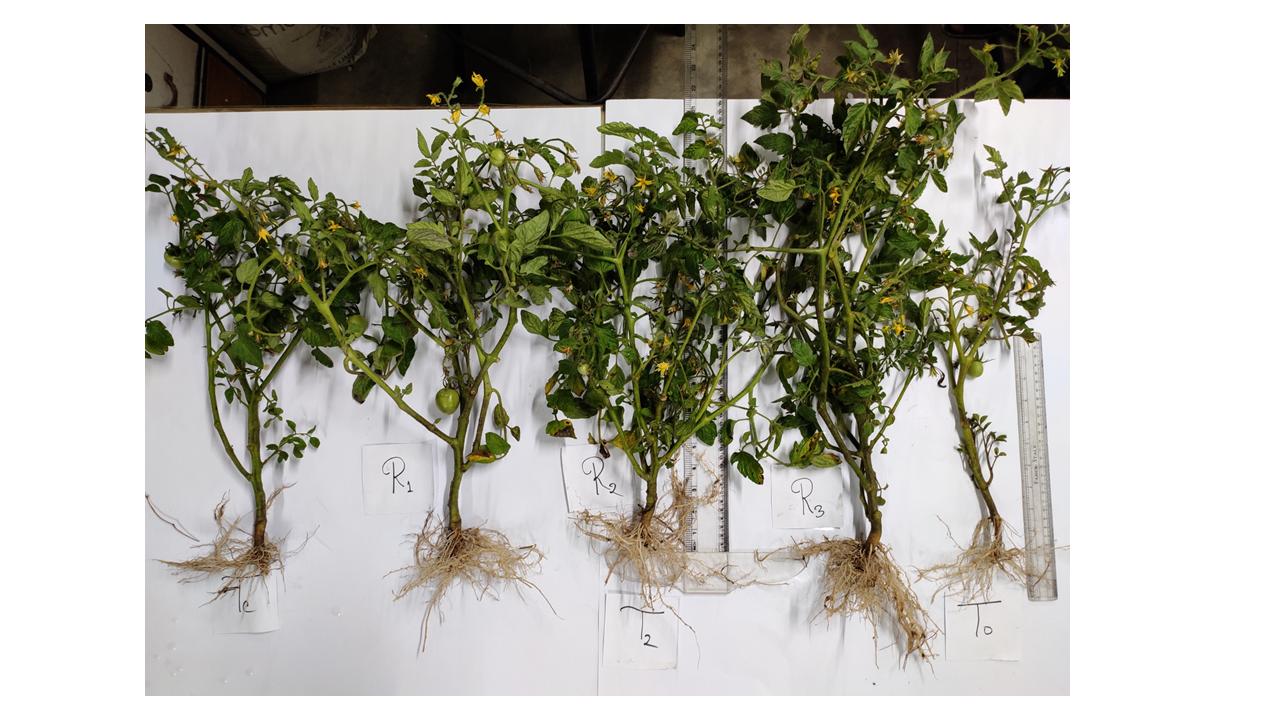

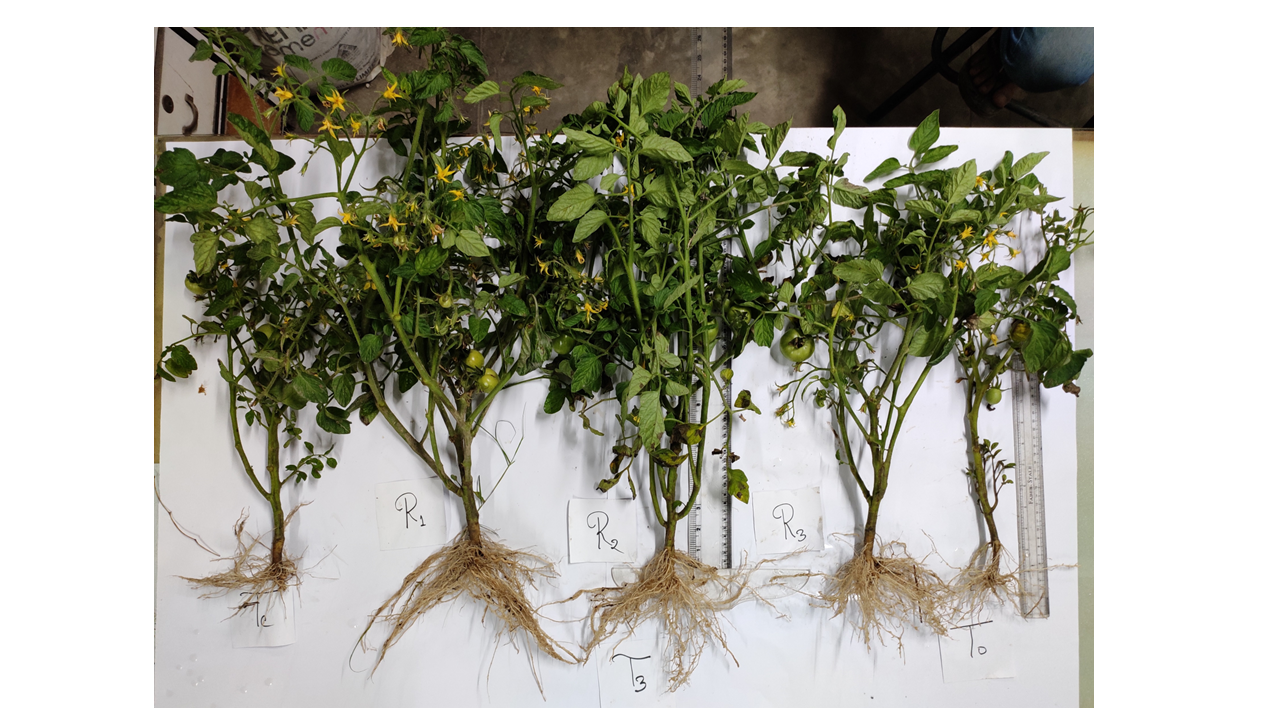


Figure S3B. Tomato plants after 45 Days of plantation.

**30% urea+ HSTU-ASh6 treatment**

**70% urea+ HSTU-ASh6 treatment**

**100% urea+ HSTU-ASh6 treatment**


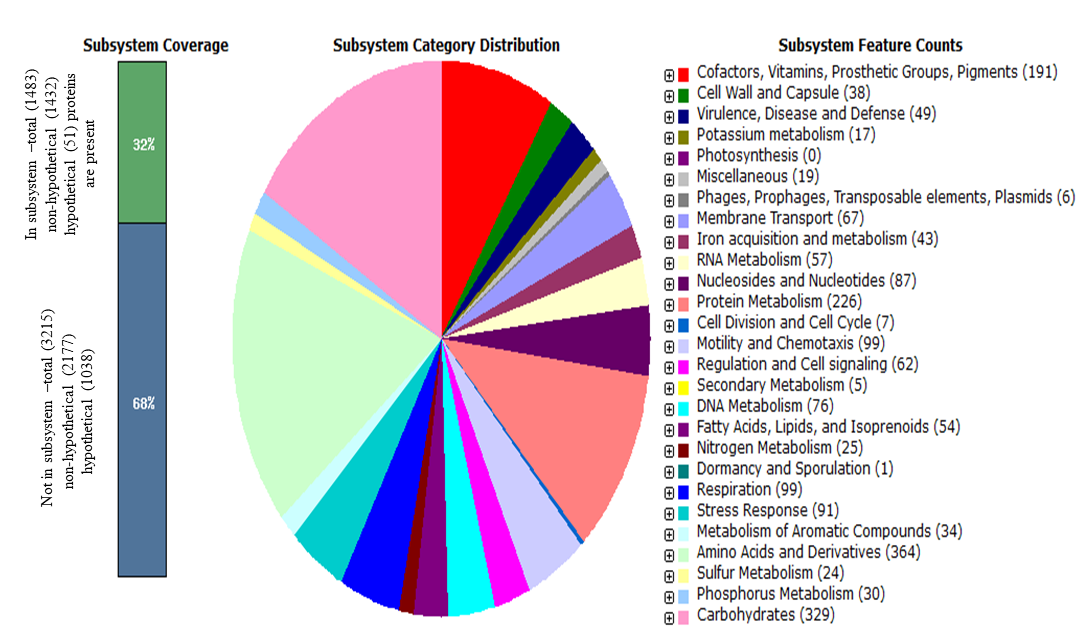


**Figure S4:** Subsystem of the RAST annotation
